# Brain-derived neurotrophic factor in Alzheimer’s disease and its pharmaceutical potential

**DOI:** 10.1186/s40035-022-00279-0

**Published:** 2022-01-28

**Authors:** Lina Gao, Yun Zhang, Keenan Sterling, Weihong Song

**Affiliations:** 1 Shandong Collaborative Innovation Center for Diagnosis, Treatment and Behavioral Interventions of Mental Disorders, Institute of Mental Health, College of Pharmacy, Jining Medical University, Jining, 272067 Shandong China; 2 Townsend Family Laboratories, Department of Psychiatry, The University of British Columbia, 2255 Wesbrook Mall, Vancouver, BC V6T 1Z3 Canada; 3 National Clinical Research Center for Geriatric Disorders, Xuanwu Hospital, Capital Medical University, Beijing, 100053 China; 4 Institute of Aging, Key Laboratory of Alzheimer’s Disease of Zhejiang Province, School of Mental Health and The Affiliated Kangning Hospital, Wenzhou Medical University, Wenzhou, 325000 Zhejiang China; 5 Oujiang Laboratory (Zhejiang Lab for Regenerative Medicine, Vision and Brain Health), Wenzhou, 325001 Zhejiang China

**Keywords:** Brain-derived neurotrophic factor, Alzheimer’s disease, Amyloid β protein, Tau protein, Neuroinflammation, Neuronal apoptosis

## Abstract

Synaptic abnormalities are a cardinal feature of Alzheimer’s disease (AD) that are known to arise as the disease progresses. A growing body of evidence suggests that pathological alterations to neuronal circuits and synapses may provide a mechanistic link between amyloid β (Aβ) and tau pathology and thus may serve as an obligatory relay of the cognitive impairment in AD. Brain-derived neurotrophic factors (BDNFs) play an important role in maintaining synaptic plasticity in learning and memory. Considering AD as a synaptic disorder, BDNF has attracted increasing attention as a potential diagnostic biomarker and a therapeutical molecule for AD. Although depletion of BDNF has been linked with Aβ accumulation, tau phosphorylation, neuroinflammation and neuronal apoptosis, the exact mechanisms underlying the effect of impaired BDNF signaling on AD are still unknown. Here, we present an overview of how BDNF genomic structure is connected to factors that regulate BDNF signaling. We then discuss the role of BDNF in AD and the potential of BDNF-targeting therapeutics for AD.

## Introduction

Alzheimer’s disease (AD) is the most common neurodegenerative disorder in the elderly [1]. AD affects 11% of the population over the age of 65 and nearly half of people aged 85 years and older. However, there is no definitive early diagnostic marker and no effective prevention or disease-modifying treatment for AD [2–4]. As reported by the AD drug development pipeline in 2020, a total of 121 agents are undergoing clinical trials [5]. Most candidate agents (80.1%) are disease-modifying therapies targeting disease onset or progression, 9.9% are symptomatic cognitive enhancers, and 10.0% are symptomatic agents addressing neuropsychiatric and behavioral changes. On June 7, 2021, the U.S. Food and Drug Administration (FDA) approved aducanumab as a disease-modifying therapy for AD under its “accelerated approval” pathway, meaning aducanumab demonstrated an effect on a surrogate endpoint that predicts it will be clinically beneficial [6]. This decision is surprising and controversial, especially since the surrogate endpoint used was the reduction of amyloid β (Aβ) rather than clinical efficacy. Moreover, the FDA's advisory committee had previously recommended against aducanumab's approval due to the insufficient evidence to support the drug to improve cognitive decline during the phase 3 trial [7, 8]. This decision to approve aducanumab as a treatment for AD is particularly alarming as it does not provide any guidance on which patients would likely benefit. There is no definitive evidence showing that removing amyloid deposits will be therapeutically beneficial for all individuals diagnosed with AD, especially for patients at more advanced stages of the disease process. Furthermore, many previous drugs targeting amyloid deposits have failed in later-stage clinical testing due to poor efficacy. As a result, there has been a growing emphasis over the past 5 years to pursue intervention strategies that target other damaging features of AD, including those that might mediate the downstream consequences of Aβ accumulation should plaque removal fail to halt disease progression. Current examples include candidate therapies that promote neurogenesis and the protection of neurons and synapses, as well as interventions that target inflammatory, vascular, or epigenetic mediators of AD pathology [5].

AD pathology is characterized by an accumulation of two aggregated proteins in the brain, Aβ and tau, leading to the formation of extracellular neuritic plaques and intracellular neurofibrillary tangles (NFTs), respectively [9]. Following Aβ and tau pathology, AD patients further exhibit synaptic abnormalities, neuronal loss, cognitive decline and memory impairments as the disease progresses [10–13]. Aβ is the central component of neuritic plaques and is a proteolytic product of the amyloid β precursor protein (APP) [14]. NFTs are formed from the hyperphosphorylated microtubule-associated protein tau. Aβ- and tau-induced neuroinflammation and neuronal apoptosis contribute to AD pathogenesis [15, 16]. AD is a complex and multifactorial disorder. Different hypotheses have been proposed to explain the pathologic process of AD, including the cholinergic hypothesis [17], the tau hypothesis [18, 19], the glutamate dysfunction hypothesis [20], the amyloid cascade hypothesis [21, 22], the inflammatory hypothesis [23], and the mitochondrial cascade hypothesis [24]. However, these hypotheses can only account for certain aspects of the disease, and the mechanism leading to AD pathogenesis remains elusive. As the cognitive impairment in AD is due to neurodegeneration, neurotrophic factors including brain-derived neurotrophic factor (BDNF) may slow the progression of neurodegeneration and serve as a promising strategy for AD intervention.

BDNF is a well-studied growth factor in the mammalian brain. It plays a vital role in facilitating nerve growth and maturation through development stages and regulating synaptic transmission and plasticity in adulthood [25, 26]. In the brain, BDNF is mainly synthesized in cell bodies of neurons and glial cells and then transported to presynaptic terminals and postsynaptic dendrites. The localization of BDNF and its receptor, tropomyosin receptor kinase B (TrkB), to glutamate synapses regulates neurotransmitter release, ion channel activity, axonal pathfinding and neuronal excitability [27]. In the context of AD, BDNF depletion is associated with tau phosphorylation, Aβ accumulation, neuroinflammation and neuronal apoptosis [28]. Stimulation of BDNF leads to tau dephosphorylation through activation of TrkB and phosphatidylinositol 3-kinase (PI3K) signaling [29, 30]. Aβ disrupts BDNF signaling through dysregulation of the glutamatergic *N*-methyl-d-aspartate receptor (NMDAR)/Ca^2+^/calpain signaling cascade [31]. Upregulation of BDNF by the extracellular regulated kinases/cyclic AMP response element-binding protein (ERK/CREB) signaling pathway can ameliorate the Aβ-induced neuronal loss and dendritic atrophy [32]. Silencing BDNF antisense RNA can significantly up-regulate BDNF, reduce Aβ-induced neurotoxicity, and enhance cell viability [33]. Growing evidence also suggests that the BDNF signaling plays a critical role in modulating the downstream consequences of Aβ accumulation in AD. BDNF mediates the link between inflammation and neuroplasticity by regulating the release of neurotransmitters (such as glutamate and gamma-aminobutyric acid) following nuclear factor-κB (NF-κB) activation [34, 35]. As the disease progresses, BDNF levels in the brain [36], blood [37] and cerebrospinal fluid (CSF) [38] of AD patients are reduced. In addition, higher serum levels of BDNF have been correlated with improved cognitive function in AD [39]. These findings have led to an increasing interest in BDNF as a potential biomarker for diagnosis of or as a therapy for AD. In the following, we will discuss the role of BDNF in AD and the pathways by which BDNF alleviates the progression of AD, highlighting the potential of BDNF-targeting therapeutics for this devastating disease.

## Overview of BDNF

### BDNF gene structure, expression and function

**Fig. 1:**
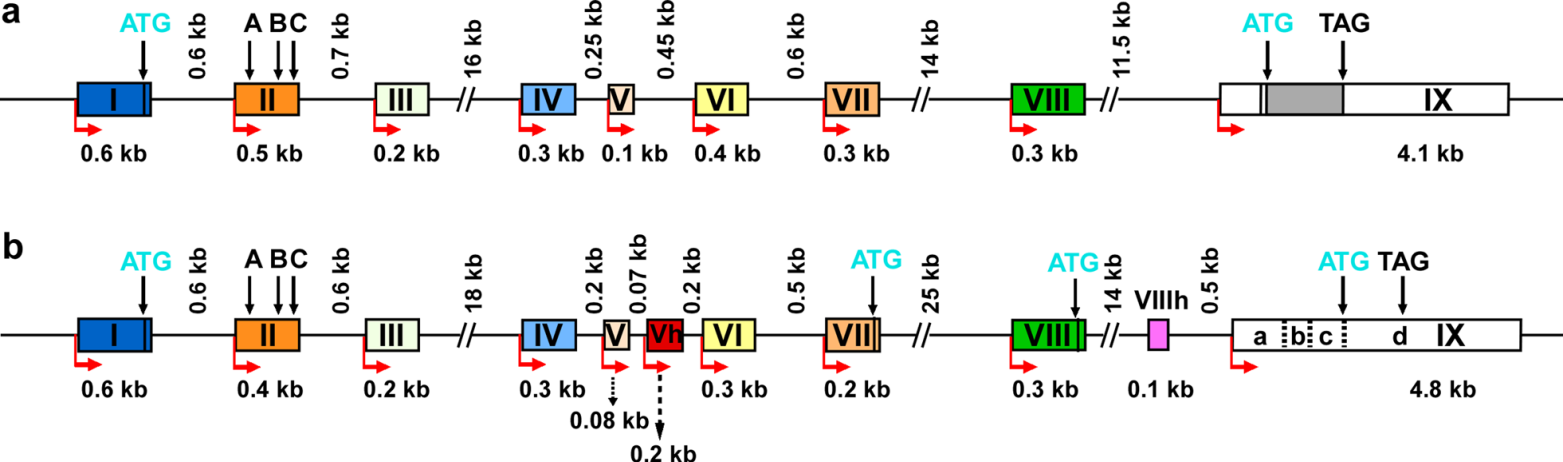
Rodent and human *BDNF* gene structures. **a** Rodent *Bdnf* gene structure. **b** Human *BDNF* gene structure. Exons are shown as boxes and introns are shown as lines. In both structures, the same color indicates that human exons and rodent exons are homologous. The different exons (Vh and VIIIh) are shown as red box and pink box, respectively. In exon II, there are three transcript variants which are marked as A, B and C. In human *BDNF* exon IX, there are four different regions that are marked as a, b, c and d. The numbers above the introns and below the exons indicate their base pair sizes. The red arrows indicate the positions in which the transcription starts. ATG represents the sites of the translational start and TAG marks the location of stop codons

BDNF has a complex gene structure and tissue-specific expression pattern. As shown in Fig. 1a, rodent *BDNF* genes consist of 9 exons and 9 individual functional promoters [40, 41]. These promoters control the expression of BDNF variants encoding the same BDNF protein. This unique genomic structure allows various factors to regulate BDNF signaling in different ways. Furthermore, each BDNF isoform can be associated with a distinct set of functional outcomes [42]. Selective disruption of BDNF expression from *Bdnf* promoter I, II, IV or VI in mutant mice (*Bdnf*-e1, e2, e4 and e6 mice) is linked with different BDNF-associated molecular and behavioral phenotypes. Compared with wild-type mice, *Bdnf*-e1 and e2 mutants show more aggressive behaviors accompanied by increased gene expressions of serotonin transporter 5-HTT (*Slc6a4*) and 5-HT2A (*Htr2a*). On the other hand, *Bdnf*-e4 and e6 mutant mice are not aggressive and show altered expression of the 5-HT receptor. Specifically, loss of BDNF from promoters IV and VI suppresses GABAergic neurotransmission, resulting in decreased expression of genes involved in peptide and hormonal signaling in the brain, including somatostatin (*Sst*), corticotropin-releasing factor-binding protein (*Crhbp*), cortistatin (*Cort*) and tachykinin (*Tac1*). Quantitative analysis of BDNF protein further showed that the individual *BDNF* transcripts have a region-specific expression pattern in the hypothalamus, prefrontal cortex, and hippocampus [42]. For example, *BDNF* promoters I and II mainly contribute to the total BDNF levels in the adult hypothalamus, while promoters IV and VI contribute more to BDNF levels in the prefrontal cortex and hippocampus.

Epigenetic changes in chromatin structure can also regulate the activity-dependent BDNF transcription. Specifically, neuronal activation is associated with increased production of BDNF and exon IV promoter activity (promoter upstream of BDNF exon IV) in mice [43]. Moreover, the transcription of *Bdnf* from the exon IV promoter is enhanced in the brains of DNA methyltransferase 1 null (Dnmt1)^−/−^ mice at embryonic day 18. This alteration may be associated with reduced CpG methylation within the *Bdnf* exon IV promoter or dissociation of the methyl-CpG-binding protein (MeCP2) and its corepressors (e.g. MecP2-histone deacetylase-mSin3A complex) from the *Bdnf* exon IV promoter [44–46].

Previous studies have shown that the regulation of BDNF at the mRNA level may affect the brain function. Two *Bdnf* mRNA transcripts that facilitate different subcellular localizations have been identified in the murine brain [47]. One transcript containing a short 3’ untranslated region (3’ UTR) is localized in the soma of hippocampal neurons, while the other transcript containing a long 3’ UTR is distributed in the dendrites. Inducing a mutation in the long 3’ UTR in mice leads to expression of a truncated version of the transcript and impairs the dendritic targeting of *Bdnf* mRNA, such that BDNF expression is shifted from dendrites to the soma. This results in deficits in the pruning of dendritic spines and selective impairment of long-term potentiation (LTP) at dendritic synapses [47]. In addition to the transcript species selectivity, *BDNF* mRNAs also display activity-dependent dendritic localization in vitro, with transcripts I and IV selectively affecting proximal dendrites and transcripts II and VI selectively affecting distal dendrites. It has also been demonstrated that the dendritic targeting of short 3’ UTR can be induced by both depolarization and NT3, via binding to cytoplasmatic polyadenylation element-binding proteins (CPEB)-1, CPEB-2, embryonic lethal abnormal vision-like proteins (ELAV)-2 and ELAV-4, while the inducible dendritic targeting of long 3’ UTR requires ELAV-1, ELAV-3, ELAV-4 and Fragile X mental retardation syndrome-related (FXR) proteins [48–50]. This suggests that specific *BDNF* variants may selectively respond to different extracellular stimuli in order to modulate neuronal development and synaptic plasticity.

It is important to note that there are remarkable differences in the regulatory mechanisms of rodent and human *BDNF* genes. As shown in Fig. 1b, the human *BDNF* gene contains 11 exons and 9 promoters [51]. The expression of the human *BDNF* gene in particular brain regions is also highly regulated at the transcription level. For example, it has been found that the amygdala has relatively high expression of *BDNF* transcripts containing exons I, IV and VI. On the other hand, the *BDNF* exon II transcript is relatively upregulated in the cerebellum, while higher expression of exon IXabcd transcripts is found in the striatum, thalamus and globus pallidus. In humans, the promoters upstream of Exons I-VIII control regional and cell-type-specific expression, and the promoter upstream of Exon IX regulates activity-dependent BDNF expression. Exons Vh and VIIIh are human-specific and are not found in rodents. Exon Vh has an upstream sequence and a separate promoter, while exon VIIIh has no independent promoter to control its expression. Thus, various BDNF transcripts can be generated by using alternative promoters and splicing mechanisms, and some of these mechanisms differ substantially for rodent and human *BDNF* genes.

Transcription of noncoding natural antisense RNAs from the *anti-BDNF* gene to the *BDNF* gene locus showed that *BDNF* and *anti-BDNF* transcripts form dsRNA duplexes in the human brain [51]. This indicates that the anti-*BDNF* transcripts play a crucial role in regulating BDNF expression. The possible roles of anti-*BDNF* may include regulating *BDNF* pre-mRNA splicing and inhibition of *BDNF* transcription or *BDNF* translation. The transcription of *BDNF* mRNA can also be regulated by Ca^2+^ influx because Ca^2+^ can initiate the binding of CREB and calcium-responsive transcription factor (CaRF) to the *BDNF* promoters [52]. Moreover, many other regulators such as basic helix-loop-helix B2 and NF-κB have been identified to bind to *BDNF* promoters [53, 54]. The multiple promoters in the *BDNF* gene mediate complex transcription mechanisms. How the different BDNF mRNA variants then respond to intracellular processes and extracellular environments will lead to the diversity of BDNF neuronal distribution and biological functions.

The full-length BDNF protein has 247 amino acids and is encoded by the *BDNF* gene on human chromosome11p13. As a secreted protein, BDNF is initially synthesized in the endoplasmic reticulum as a precursor protein, called pre-pro-BDNF, which is cleaved into the pro-BDNF isoform (~ 32 kDa) when translocated to the Golgi apparatus. There are three fates of pro-BDNF: (1) intracellular cleavage by furin or convertases followed by release of mature BDNF (mBDNF) (~ 14 kDa); (2) secretion as pro-BDNF and extracellular cleavage by metalloproteinases 2 (MMP2), MMP9 and plasmin; (3) secretion as pro-BDNF without modification [55–58]. The cleavage conversion of pro-BDNF is controlled by  tissue plasminogen activator (tPA) [59]. BDNF functions are subsequently initiated by binding to one of its receptors, such as TrkB and p75 neurotrophin receptor (p75^NTR^) [60]. Notably, the balance of pro-BDNF and mBDNF is important for synaptic plasticity. Pro-BDNF binds specifically to p75^NTR^ to regulate cell death and long-term depression (LTD) [26, 61–63], while mBDNF binds more readily to TrkB to promote cell survival and LTP [64, 65]. As a co-receptor, sortilin is also involved in pro-BDNF-induced apoptosis [66, 67]. The binding region of pro-BDNF-sortilin interaction is located within amino acid residues 71–100 [68]. Therefore, the distinct binding affinities of the BDNF isoforms to various receptors are closely correlated with their action on synaptic plasticity. As a portion of pro-BDNF, BDNF pro-peptide is generated through N-terminal cleavage of pro-BDNF. The BDNF pro-peptide is localized at presynaptic termini to enhance hippocampal LTD [26, 69] and regulate dendritic spine morphology [70]. Therefore, the mBDNF, pro-BDNF and BDNF pro-peptide all modulate synaptic functions in the brain.

BDNF serves many important functions in the adult brain and has been shown to play a critical role in supporting neuronal survival and differentiation [71], enhancing synaptic transmission [72] and synaptic plasticity [73], and promoting memory processes [71, 74]. The neurotrophic functions of BDNF are primarily mediated by the TrkB receptor [75]. BDNF and TrkB are present at both presynaptic and postsynaptic sites in neurons. Presynaptic BDNF promotes neurotransmitter release (e.g. glutamate and GABA) via the TrkB–MAP kinase–synapsin signaling cascade [76]. It has been reported that myosin VI (Myo6) and Myo6-binding protein (GIPC1) can form a complex to engage TrkB, which may be necessary for the BDNF–TrkB-mediated presynaptic function and synaptic plasticity [75]. Postsynaptic BDNF signaling contributes to enhancing the function of various ion channels, such as NMDAR, as well as calcium, sodium and potassium channels [77, 78]. Once activated, the synaptic effects of BDNF signaling occur within seconds [79]. Maintaining the functional regulation of the BDNF/TrkB system is vital to healthy ageing, as the loss of BDNF signaling in the adult brain has been associated with impaired learning and memory [80], declining cognition [81], and abnormal mood-related behavior [82].

### Distribution of BDNF

BDNF mRNA is distributed throughout the central nervous system (CNS), including the cortical, hippocampal, nigral, amygdala and thalamic regions [83–85]. The highest level of BDNF mRNA is found in the hippocampus [86]. Hippocampal BDNF expression is primarily localized to the CA2, the medial portion of CA1, and the nuclei of granule cells in the dentate gyrus and the pyramidal cell layer [87]. In addition, BDNF is highly produced and expressed in the entorhinal cortex, a key brain area for learning and memory and a major relay between the cortex and hippocampus. It has been found that BDNF produced in the entorhinal cortex is actively transported to the hippocampus [88]. The mRNA expression of BDNF has also been detected in the granule cell layer of the cerebellum [86]. Notably, although BDNF mRNA expression is lacking in certain regions of the brain (e.g., the adult rodent striatum), substantial amounts of BDNF protein can be found in these regions because axons can anterogradely transport BDNF mRNA to the terminals of BDNF-expressing neurons [85, 86]. Thus, factors regulating the neuronal circuitry between brain regions that contain BDNF-producing neurons (i.e., the entorhinal cortex) and regions that lack BDNF-producing neurons (i.e., the hippocampus) play a critical role in governing BDNF trafficking in the brain. Another important source of BDNF in the body is platelet cells [89]. Peripheral BDNF is stored in blood platelets and synthesized by vascular cells, epithelial cells, muscle cells, leukocytes and macrophages [90, 91]. Pro-BDNF was found in human blood samples with a molar ratio (of pro-BDNF to BDNF) of 1:5 in platelets and 10:1 in plasma. Platelet activation was also found to selectively release BDNF, but not pro-BDNF [92]. Recently, BDNF was also found to promote platelet activation, aggregation and secretion by activating a truncated form of the TrkB receptor [93]. However, assessments of BDNF levels in platelets have not been fully examined in AD patients. Questions about the role of pro-BDNF in platelet function and how the platelet ratio of pro-BDNF/BDNF related to neuronal levels remain unanswered.

### Methods of BDNF detection

Several commonly used techniques and novel approaches have been reported for detecting BDNF levels [94]. *BDNF* gene expression is commonly measured by reverse-transcription polymerase chain reaction (RT-PCR) or quantitative real-time PCR (qPCR) [95]. While this technique is very sensitive, different cell types have unique transcriptomes and thus may possess distinct regulatory mechanisms. More recently, single-cell transcriptomic analysis has attracted great interest as a means to provide more accurate information on how individual cells respond to signals or when they acquire abnormal phenotypes [96–98]. In previous research, the expression profile of BDNF/TrkB has been studied in various cell types and diseases by combining single-cell transcriptome analysis with overexpression, knockout, or knockdown of TrkB [99–102]. These studies clarified whether the protective mechanism of BDNF on neuronal survival or neurogenesis is mediated via TrkB; and the targets and effects of BDNF anterogradely transported from the cortex to other regions of the brain (such as striatum). Recently, a qRT-PCR protocol with HEX (hexachloro-fluorescein) and FAM (6-carboxyfluorescein) to detect the products of Val66- and Met66-coding *BDNF* allele has been developed for detection of *BDNF* Val66Met polymorphism [103].

Levels of the BDNF protein in brain tissues, blood, CSF and saliva can also be detected by sandwich enzyme-linked immunosorbent assay (ELISA) [104]. There are four different types of commercial ELISA kits available for BDNF [105], including (1) kits designed to recognize pro-BDNF or mBDNF selectively; (2) antibodies against the carboxy-terminal of mBDNF; (3) monoclonal antibodies against mBDNF; and (4) monoclonal antibodies against recombinant mBDNF. The first class of ELISA kits are highly selective for each target, although the sensitivity to pro-BDNF in these kits is 0.5 ng/ml, much lower than mBDNF (5–8 pg/ml). Thus, it is not easy to achieve accurate detection of pro-BDNF in body fluids using this method. The last three routinely used kits recognize both pro-BDNF and mBDNF. Given the divergent biological functions of pro-BDNF and mBDNF, highly sensitive ELISA kits must be developed to differentiate between the BDNF isoforms. Notably, Bockaj and colleagues recently demonstrated a fast and reliable method for point-of-care quantification of circulating BDNF levels that could potentially function as a diagnostic tool [106]. Briefly, they developed a device (EndoChip) capable of detecting BDNF using only small amounts of blood collected through a finger prick. The device is a polymer-based chip with nanopores and a wrinkled gold film (electrode/sensing layer). An increase in BDNF concentration (0.1–2.0 ng/ml) causes remarkable differences in redox current. Alternatively, the levels of BDNF in brain tissues, cell lysates and media of cultures have been measured by immunoprecipitation/western blot analysis, which can clearly distinguish between pro-BDNF and mBDNF [107, 108]. A reliable measurement of low levels of endogenous pro-BDNF can also be obtained by designing monoclonal antibodies specific for the pro-domain [109].

Techniques such as confocal microscopy are used to visualize the expression, secretion, and trafficking of BDNF. As a practical example, Sindbis viral infection of hippocampal neurons has previously been used to enable cultured neurons to selectively express constructs containing either valine BDNF (valBDNF) or methionine BDNF (metBDNF), followed by GFP [110]. Visualizing valBDNF-GFP or metBDNF-GFP fluorescence via confocal microscopy could then be used to identify the effects of these single nucleotide polymorphisms (SNP) on the expression, distribution, intracellular trafficking and activity-dependent secretion of BDNF in living neurons. While confocal microscopy provides excellent spatial resolution, it is not well suited for investigating real-time dynamic processes. As an alternative, lentivirus encoding BDNF-pHluorin, a reporter composed of full-length (pro)BDNF and a pH-sensitive form of GFP, has also been used to investigate dynamic biological events such as the secretion of BDNF in primary cortical neurons [111, 112]. As a drawback, it is difficult to detect the reporter gene in intracellular vesicles because of the low pH in the lumen. Deacidification causes a rapid enhancement in fluorescence during exocytosis, which then decays because the cargo diffuses into the extracellular medium [111]. To address this, the spatiotemporal dynamics of BDNF exocytosis can be monitored using total internal reflection fluorescence time-lapse microscopy. A *Bdnf*-Luciferase transgenic mouse model was also generated for high-throughput screening of candidate agents that activate endogenous BDNF expression in cultured primary cortical neurons [113, 114]. Taken together, the recent advances in these methods may help to examine further the transcription, translation, expression, secretion, transportation, biological function and therapeutic potential of BDNF in AD.

## The role of BDNF in AD

### Animal studies

Different animal models are used to dissect many of the molecular and cellular mechanisms that drive the pathogenesis of AD. Currently, the most popular approaches employ various transgenic rodent models that exhibit amyloid and tau pathologies, such as Tg2576 [115], APP and presenilin 1 (APP/PS1) [116], Tau/APP [117], J20 [118], 3× Tg [119] and 5× FAD [120] transgenic mice, as well as McGill-R-Thy1-APP [121] transgenic rats. Similarly, these transgenic models have been previously used to investigate how the expression and regulation of BDNF are altered in the context of AD-like pathologies, and how intervention strategies or therapeutic agents that enhance BDNF could serve as a potential treatment for AD [117, 122, 123]. For example, previous studies have shown that APP/PS1 transgenic mice that express the mutated variant of human APP and PSEN1 genes linked to familial AD, namely, the Swedish APP KM670/671NL mutation (APPswe) and PSEN1 L166P mutation, exhibit memory deficits and impaired hippocampal neurogenesis in adulthood [124]. Facilitating social interaction by housing APP/PS1 mice with wild-type mice reverses the deficits in memory and neurogenesis, an effect that can be mimicked by overexpressing BDNF or blocked by ablating it. Gene delivery or overexpression of BDNF has also been shown to enhance hippocampal LTP and inhibit the effect of Aβ and tau on cell loss [88, 125, 126]. Furthermore, BDNF treatment decreases the generation of toxic Aβ by promoting the α-secretase processing of APP in transgenic APP/PS1 mice, suggesting it may be able to modulate the amyloidogenic pathway directly [127].

In loss-of-function experiments, triple transgenic APP/PS1/BDNF^+/−^ mice exhibited an earlier onset of learning deficits and accelerated impairment in a two-way active avoidance task compared with APP/PS1 or BDNF^+/−^ mice [128]. However, no change in plaque density was observed between APP/PS1 and APP/PS1/BDNF^+/−^ mice [128]. Similarly, by crossing BDNF^+/−^ mice with APPdE9 mice (bearing APPswe and PSEN1ΔE9 mutations), researchers found that while the haploinsufficiency-induced decrease of BDNF impaired learning and memory, it did not alter amyloid pathology [129]. Aged triple transgenic mice (3× Tg, bearing APPswe, MAPT P301L, and PSEN1 M146V mutations) have widespread Aβ plaques and neurofibrillary tangles [119]. Knockdown of BDNF in the aged 3× Tg/BDNF^+/−^ mice led to a significant reduction of BDNF levels, but this did not appear to exacerbate Aβ and tau pathology [130]. These results suggest that chronically reduced expression of *BDNF* does not affect Aβ and tau pathologies. On the other hand, Wang et al. reported that deprivation of BDNF/TrkB indeed contributes to AD-like pathologies in wild-type mice [28]. Several possible causes may contribute to these conflicting results. First, there may be inherent differences in the animal models themselves. For example, compensatory processes may have occurred to respond to the chronically depleted levels of BDNF in the transgenic models. Second, decreased BDNF expression may reduce APP expression [131]. Third, there may be a dose-sensitivity window whereby the degree of BDNF knockdown could have a differing effect on Aβ or tau pathologies. Lastly, BDNF may target the cellular and molecular pathologies downstream of Aβ accumulation when exerting its therapeutic effects.

### Clinical investigations

The first report on BDNF from studies in a clinical population came from Phillips and colleagues who found that BDNF mRNA was reduced in postmortem hippocampal samples obtained from AD patients, suggesting that BDNF may have contributed to the progressive atrophy of neurons in AD [132]. Similar reductions in BDNF mRNA levels have been found in samples from the parietal cortex and entorhinal cortex of AD patients [133, 134]. Other reports have suggested that the decreased BDNF protein in the hippocampus, temporal cortex, and CSF in AD may correlate with the degeneration of specific neuronal populations, such as the basal forebrain cholinergic system [135–137]. Reduced levels of both pro-BDNF and mBDNF also occur early in the progression of AD [36]. However, it should be noted that although decreased BDNF levels in brain tissues have been associated with AD progression, there have been conflicting reports on whether BDNF levels are reduced in the CSF of AD patients. These conflicting results may be caused by a few different factors. First, most clinical studies have analyzed total BDNF concentrations by ELISA, which cannot reliably differentiate pro-BDNF from mBDNF. Second, the lower threshold for detection must be increased as there is a low baseline level of CSF BDNF [138]. Third, CSF BDNF levels also decrease during healthy aging, suggesting this may only serve as a prognostic biomarker for younger individuals with an elevated risk of developing AD [137]. These limitations should be addressed before BDNF is used as a promising biomarker for AD diagnosis in the clinical setting.

Efforts to determine whether plasma BDNF levels can serve as a blood-based biomarker in AD have received increasing attention over the past decade [139–141]. Blood sample collection is minimally invasive and far more suitable for detecting and monitoring AD pathologies in healthcare settings than existing methods that require CSF or PET analyses. However, previous studies on plasma BDNF levels in AD patients have conflicting results. While some studies reported that the peripheral BDNF levels in AD patients were decreased [138, 142–144], others found no difference or even enhanced BDNF concentrations in AD patients [145–147]. Many meta-analyses have been performed to systemically analyze the change of peripheral BDNF during the development and progression of AD. It has been reported that patients with AD have significantly lower peripheral blood BDNF levels than healthy controls [148]. A higher serum BDNF level has also been linked to a reduced risk of dementia [149]. When compared with the age- and sex-matched healthy controls, blood BDNF levels initially increase during the early stages of AD and then reduce in patients with moderate or severe AD [150]. The initial increase in blood BDNF levels could be caused by compensatory repair mechanisms that arise during the early stages of AD. Then, as the severity of the disease progresses (such as Mini-Mental State Examination [MMSE] score < 20), these compensatory mechanisms may begin to fail, resulting in decreased peripheral blood BDNF levels. The association between serum BDNF and AD progression has been linked to the rate of cognitive decline. Decreased serum BDNF levels are specifically associated with fast cognitive decline in AD patients (that is, a lower MMSE score > 4 per year), rather than slow cognitive decline [140]. The association also occurs between the serum pro-BDNF levels and the hippocampal pro-BDNF levels, which are related to the hippocampal pTau expressions [151].

The evidence from clinical investigations suggests that BDNF could act as a biomarker and therapeutic target in AD. However, several key questions remain to be answered. First, how do factors associated with altered peripheral BDNF levels and AD risk (i.e., age, lifestyle, and comorbid physical conditions) modulate plasma BDNF levels as the disease progresses? Answers to these questions could provide insights into the diagnostic value of peripheral BDNF and open up the door for personalized therapeutic strategies. Second, what factors must be considered when measuring plasma BDNF concentrations? For example, BDNF concentration in serum is over 100-fold higher than plasma concentrations due to the degranulation of platelets during the clotting process [90, 91, 152]. BDNF levels in the peripheral blood are also known to be regulated by other cells such as mononuclear and epithelial cells [153], and these regulatory mechanisms may be altered under certain conditions that could obscure any findings. Third, would the diagnostic validity of plasma BDNF levels be improved when combined with other blood-based biomarkers? Some researchers proposed composite biomarkers (i.e., serine/threonine kinase, DYRK1A, BDNF, and homocysteine) to identify AD at an early stage [154].

### Genetic evidence

Certain *BDNF* gene polymorphisms have a significant impact on hippocampal function and memory. The dbSNP: rs6265 SNP in the human *BDNF* gene is a common functional nucleotide polymorphism that leads to a methionine (Met) substitution for valine (Val) at codon 66 (Val66Met, G196A) [155]. The substitution of Val by Met modulates both the intracellular trafficking of pro-BDNF and the secretion of mBDNF [110, 156]. Further insight into this mechanism comes from studies demonstrating that the Val66Met SNP impairs the dendritic trafficking of BDNF mRNA by disrupting interaction of BDNF with translin [157] and disturbing the intracellular sorting and secretion of BDNF by blocking its interaction with sortilin [158].

Several lines of evidence have shown that the *BDNF* Met_66_ allele exacerbates Aβ-dependent AD pathogenesis and adversely impacts hippocampal function and human episodic memory [110, 159–162]. Since the *BDNF* Val66Met has no relationship with the rates of change in cognitive decline among healthy adults with low Aβ, it has been proposed that high Aβ levels coupled with Met_66_ carriage may be used as prognostic markers in the preclinical stage of AD [163]. Further support comes from studies showing that the *BDNF* Val66Met polymorphism decreases the hippocampal–medial prefrontal connectivity, increases the vulnerability of the memory network to Aβ, and worsens cognitive decline [164]. Among the elderly with normal cognition, those who carry *BDNF* Val66Met will experience faster cognitive decline and greater hippocampal atrophy [165]. APOE is a risk factor for late-onset AD. MCI patients carrying both the *APOE* ɛ4 and *BDNF* Met alleles exhibit more obvious memory deficits, though no significant changes in brain structure are observed [165]. Moreover, the *BDNF* Met_66_ allele is associated with increased CSF concentrations of total tau and increased pTau concentrations in mutation carriers [159].

Many findings suggest that the *BDNF* Met_66_ allele may exacerbate AD-related pathologies. However, studies examining this relationship more closely suggest that this association may depend on the severity of the disease and the sex of the individual. It has been reported that the Met_66_ allele increased AD risk in females but not in males, suggesting that BDNF may be a sex-specific risk factor for AD [166–168]. Additionally, the transition from healthy cognition to cognitive impairment in AD can be characterized as a progression from subjective cognitive decline (SCD) during the preclinical stages to mild cognitive impairment (MCI) during prodromal stages, and then to dementia during the clinical stages of the disease. The Val66Met polymorphism increases the risk of progressing from SCD to MCI, and from MCI to AD, exclusively in women. The Met allele also diminishes the transition time from SCD to MCI [169]. Therefore, the influence of Val66Met polymorphism on AD varies by both sex and disease severity (or stage of the disease). Furthermore, the reduced levels of BDNF protein in the temporal cortex of AD patients are suggested to have no association with BDNF polymorphisms [135]. Genome-wide association studies of AD have similarly shown that the *BDNF* Val66Met is not a risk factor for AD [170]. These findings suggest that the *BDNF* Val66Met polymorphism may interact with events downstream of AD pathogenesis, accelerating the progression of dementia in a subset of patients.

Ultimately, there are conflicting results regarding the association between the *BDNF* Met_66_ allele and AD-related risk and pathologies. Differences in these findings may arise because the targeted phenotypes of these studies are different, and the BDNF gene mainly manifests in the early stages of AD. Other factors may influence the role of *BDNF* Val66Met polymorphism in AD, including ethnicity, age and sex. The *BDNF* Val66Met has linkage disequilibrium with other BDNF polymorphisms, such as C270T (rs2030324) and G712A, which may affect their interactions and downstream phenotypes, and participate in the occurrence and development of AD [171, 172]. Altogether, though the current studies do not identify that mutations in the BDNF gene are a risk factor for AD, substantial evidence supports the notion that BDNF may be a potential target for AD therapy. The association between BDNF Val66Met polymorphism and AD risk should be further examined in future studies.

## Potential mechanisms underlying BDNF’s effect on AD

### Neuronal protective effects

Neurotrophins such as BDNF play an essential role in maintaining a functional nervous system in both healthy and diseased states. Under physiological conditions, the processing from pro-BDNF to mBDNF is important for neuronal development, neuronal survival, and synaptic plasticity. The mBDNF and its receptor, TrkB, are widely expressed in the developing and adult mammalian brains [173, 174]. The pathways associated with changes in neuronal excitability are triggered by the binding of mBDNF to TrkB, indicating that TrkB activation is crucial for controlling the survival, morphogenesis, and plasticity of neurons [175]. Moreover, mBDNF/TrkB elicits many other downstream intracellular signaling pathways, such as mitogen-activated protein kinase/extracellular signal-regulated protein kinase (MAPK/ERK), PI3K, and phospholipase C_γ_/protein kinase C (PLC_γ_/PKC) [175–177]. These signaling pathways are associated with activation of the transcription factor CREB that mediates the transcription of genes essential for synaptic plasticity [175]. For example, the BDNF/TrkB signaling-mediated hippocampal LTP is dependent on the recruitment of PLC_γ_, followed by phosphorylation of calcium/calmodulin kinase IV (CaMKIV) and CREB [176]. In turn, the expression of BDNF is modulated partially by the phosphorylation of CREB in a Ca^2+^-dependent manner [178]. Additionally, there is a Ca^2+^ response element (CRE) in the *BDNF* gene to mediate BDNF transcription. In postsynaptic neurons, Ca^2+^ influx promotes phosphorylation of CREB through binding to CRE, resulting in the activation of BDNF transcription [178]. BDNF transcription in these neurons is at least partially CREB-dependent, as mutation of CRE or blockade of CREB function leads to a massive loss of BDNF transcription [178].

**Fig. 2:**
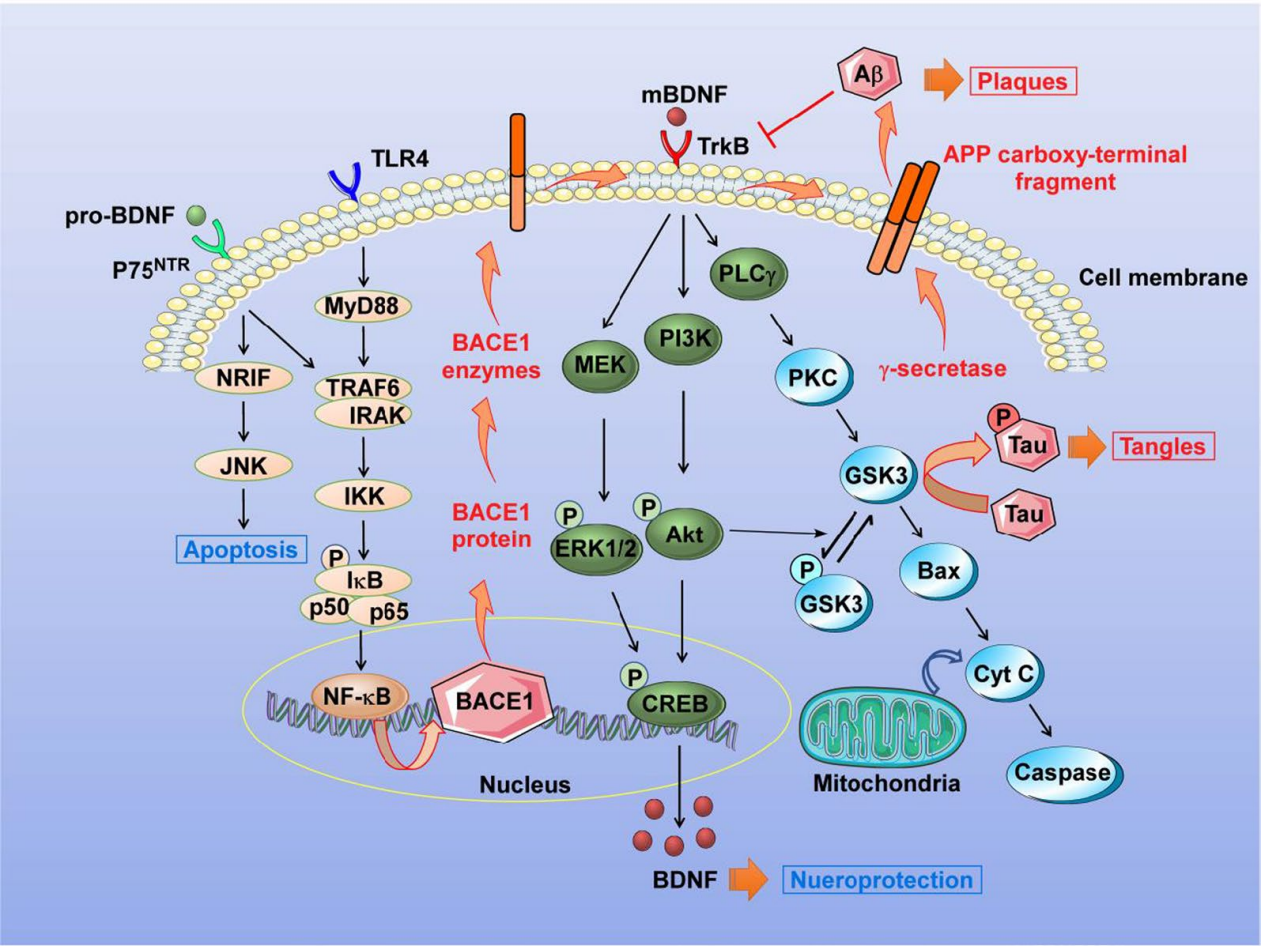
BDNF-related signaling pathways in AD. The pathways related to neuronal excitability are triggered by the interaction between BDNF and TrkB, inducing its dimerization and autophosphorylation of tyrosine residues in the cytoplasmic kinase domain. MEK, PI3K and PLCγ signaling pathways are activated to phosphorylate the transcription factor CREB that mediates transcription of genes essential for synaptic plasticity. GSK3 becomes inactive after phosphorylation, resulting in synthesis of glycogen in the liver cells. When GSK3 remains in its active form, it hyper-phosphorylates tau protein in nerve cells, resulting in the microtubule destabilization and neurofibrillary tangle formation and finally leads to AD. GSK3 also induces the overexpression of Bax to mediate apoptotic injury. Additionally, interaction between pro-BDNF and p75^NTR^ induces apoptosis through the JNK cascade. The activated NF-κB promotes the expression of β-secretase 1 (BACE1) gene, followed by the overexpression of BACE1 protein and enhanced BACE1 enzyme activity. Aβ is generated from APP by two enzymes: β-secretase (BACE1 is the major one) cuts APP first to produce a C-terminal fragments (CTFs), including C89 and C99. C99 is a membrane bound product. Then γ-secretase (including presenilin, nicastrin, APH-1 and PEN-2) cleaves C99 at a position inside the cell membrane to generate the mature Aβ peptide. In turn, Aβ inhibits the expression of TrkB, leading to neurodegeneration. BDNF: brain-derived neurotrophic factor, p75^NTR^: p75 neurotrophin receptor, TrkB: tropomyosin receptor kinase B, Aβ: amyloid β, APP: amyloid β precursor protein, BACE1: β-secretase 1; NRIF: NT receptor interacting factor, JNK: c-Jun N-terminal kinase, TRAF6: TNF receptor associated factor 6, IRAK: Interleukin-1 receptor-associated kinase, IKK: inhibitor of nuclear factor kappa-B kinase, IκB: inhibitor of NF-κB, NF-κB: nuclear factor-κB, TLR4: Toll-like receptor 4, MyD88; Myeloid differentiation primary response gene 88, TNF-α: tumor necrosis factor-α, MEK: mitogen-activated protein kinase kinase, ERK1/2: extracellular signal-regulated protein kinase 1/2, CREB: cAMP-response element binding protein, PI3K: phosphoinositide 3-kinase, Akt: protein kinase B, PLCγ: phospholipase C_γ_, PKC: protein kinase C, GSK3β: glycogen synthase kinase-3β, Cyt C: cytocheome C

Under pathological conditions such as AD, BDNF is involved in Aβ accumulation, tau phosphorylation, neuroinflammatory response and apoptosis (Fig. 2). As discussed previously, AD-related deficits in memory processes are associated with reduced BDNF levels at the synapses. Specifically, Aβ has been shown to impair the processing of BDNF in both an activity-dependent and an activity-independent manner. While Aβ reduces the activity-dependent BDNF transcription by impairing CREB phosphorylation, Aβ-stimulated reductions in basal BDNF levels are associated with a decrease of CREB transcription [179]. This may be because that CREB phosphorylation alone is not sufficient to cause BDNF induction. CREB family member works cooperatively with other transcription factors, such as CaRF [52] and myocyte enhancer factor 2 (MEF2) family members [180], to mediate BDNF transcription. Further knowledge will be needed to characterize the mechanisms in depth.

### Inhibition of tau phosphorylation

NFTs formed by hyperphosphorylated microtubule-associated protein tau are one of the neuropathological hallmarks of AD. In primary neurons and AD animal models, the overexpression or hyperphosphorylation of tau decreases BDNF expression, and in turn, BDNF regulates the expression, phosphorylation and distribution of tau [181, 182]. Overexpression of human tau in hTau (heterozygous mouse tau-knockout) and 8c-het (homozygous mouse tau-knockout) transgenic mice dramatically reduces the BDNF level [181]. Overexpression of Aβ in APP23 mice results in a reduction of BDNF mRNA, while APP23 × Tau-knockout mice show rescued BDNF levels and have no significant difference from the non-transgenic group [181]. These results indicate that overexpression of tau is responsible for BDNF down-regulation, and knockout of tau may rescue BDNF levels. To clarify the interaction between BDNF and tau, Xiang et al. demonstrated that BDNF depletion promotes tau proteolytic cleavage by provoking δ-secretase activation [183]. The subsequently generated tau N368 fragment binding to the TrkB receptor C-terminal tail, a site of PLC-γ1 binding, antagonizes BDNF/TrkB neurotrophic signaling and induces neuronal cell death. Furthermore, deprivation of BDNF/TrkB promotes phosphorylation of the Janus kinase 2/signal transducer and activator of transcription 3 (JAK2/STAT3) pathway and activation of CCAAT/enhancer-binding protein β/asparagine endopeptidase (C/EBPβ/AEP), resulting in the expression of δ-secretase [28]. In the Tau-P301L transgenic zebrafish model, significant down-regulation of BDNF is observed, which occurs in a TrkB receptor-independent manner at as early as 48 h after the Tau-P301L zebrafish embryos are fertilized [184]. BDNF knockdown leads to defective axonal development and neuronal cell death, which can be rescued by exogenous BDNF treatment. In Tau-P301L larvae, however, supplementation of exogenous BDNF repairs primary axonal growth and motility, but it does not prevent neuronal apoptosis. Treatment with a TrkB agonist, 7,8-dihydroxyflavone, completely rescues the locomotor phenotype of Tau-P301L larvae. Accordingly, reduction of BDNF is an early consequence of tau-induced neurotoxicity, and that the BDNF/TrkB signaling is necessary to protect against the tau-induced neurodegenerative effects. Furthermore, long-term treatment strategies targeting BDNF or TrkB may provide additional protection against neuronal loss and cell death. The pro-BDNF is also associated with the occurrence and development of AD. First, the pro-BDNF level in AD cortices is lower than that in healthy controls, which is consistent with the report from Peng et al. [36]. Second, the reduced expression of hippocampal TrkB receptors is linked to higher p-tau levels. Third, higher serum levels of pro-BDNF are correlated with lower pro-BDNF and higher p-tau in the hippocampus [151]. Thus, the total BDNF, mBDNF, pro-BDNF and TrkB receptors are closely associated with tau pathology, and more extensive studies are required to better understand the mechanisms linking BDNF/TrkB signaling to tau pathology, including the role of each BDNF isoform in different diseases and in various tissue specificities.

GSK3 is a key molecule linking BDNF to tau. As shown in Fig. 2, the effect of BDNF on GSK3 activity has been evaluated in the Akt and PKC signaling pathways. After BDNF binds to TrkB, the downstream PI3K is activated, followed by phosphorylation of Akt, which further phosphorylates GSK3α and GSK3β to inactivate the GSK3 proteins [185]. In addition, GSK3 phosphorylation is PKC-dependent. Inhibition of GSK3 increases BDNF mRNA and protein levels in cultured cortical neurons [186]. The biological activity of tau is modulated by its degree of phosphorylation. GSK3β acts as a critical kinase for tau protein phosphorylation [187]. It has been reported that the full-length GSK3β (47 kDa) is significantly decreased, and truncation of GSK3β (41 kDa) is markedly increased in the AD human brain when compared with healthy control cases [188]. The GSK3β truncation is positively correlated with the site-specific phosphorylation of tau (including Ser199, Thr202, Thr205, Thr212, Thr217, and Ser396). The mechanism is that excitotoxic conditions lead to a Ca^2+^-induced overactivation of calpain I, which cleaves GSK3β at Ser381-Ser382, resulting in enhanced kinase activity and the subsequent phosphorylation of tau proteins [188]. These results indicate that increasing GSK3β expression will decrease BDNF mRNA levels, and that enhancing GSK3β enzyme activity will promote tau phosphorylation. However, some conflicting results question the efficacy of BDNF as a mediator of tau phosphorylation. In tau-mutant P301L transgenic mice, the *BDNF* gene delivery attenuates cognitive deficits, promotes synaptic degeneration, but has no effect on tau hyperphosphorylation or the activity of tau-related enzymes, including GSK3β and phosphatase PP2A [189]. Inherent differences between the types of experimental models may partially account for the contradictory findings. Phosphorylated tau may quickly respond to BDNF supplementation in vitro. However, in vivo BDNF treatment is a long-term process. Further studies are required to examine the mechanism of BDNF on tauopathies in humans and animal models.

### Reduction of Aβ generation

Aβ is generated from proteolytic cleavage of APP through the amyloidogenic pathway [190–193]. Under physiological conditions, APP is predominantly cleaved via the non-amyloidogenic pathway, which occurs by α-secretase cleavage to generate the soluble αAPP fragment (sAPPα) and the membrane-anchored C-terminal fragment (CTF) C83. C83 is then cleaved by γ-secretase, resulting in the release of the nontoxic P3α fragment and CTFγ [194–197]. APP can also be cleaved by β-secretase (BACE1) at the Glu11 site or by θ-secretase (BACE2) to produce C89 and C80, respectively, precluding Aβ generation [193, 198–200]. Alternatively, APP undergoes amyloidogenic cleavage by BACE1 at the Asp1 site to release sAPPβ and C99. Next, γ-secretase cleaves the C99 to release toxic Aβ_1-40_ or Aβ_1-42_ [201].

Experimental studies suggest that Aβ deposition is closely associated with the loss of BDNF. Intracerebroventricular injection of Aβ_1-42_ oligomers downregulates BDNF mRNA and protein expression [202]. The Aβ oligomers impair the axonal BDNF retrograde trafficking, thereby adversely impacting BDNF signaling and synaptic function [203]. Oligomeric Aβ_1–42_ stimulation also significantly reduces the overall expression of *BDNF* by specifically downregulating BDNF transcripts IV and V [204]. In turn, the interruption of BDNF signaling triggers hippocampal amyloidogenesis by promoting the accumulation of PS1 N-terminal catalytic subunits, APP C-terminal fragments, and abnormal aggregation of Aβ [205]. Moreover, full-length TrkB modulates APP levels by increasing APP transcription [206]. In turn, BDNF can regulate the surface expression of full-length TrkB in a time-dependent manner. This effect was first demonstrated in hippocampal and neuronal cultures, where the level of TrkB on the plasma membrane was found to initially increase following treatment with BDNF (within seconds) and then decrease following prolonged treatment (minutes to hours) [207].

The BDNF/TrkB signaling can directly modulate APP processing. For example, retinoic acid increases the expression of TrkB in neuronal cultures [208]. Combining retinoic acid treatment with BDNF shifts APP processing to α-secretase, promoting the release of sAPP. Similarly, treating APP/PS1 mice with BDNF decreases the generation of toxic Aβ by promoting the α-secretase processing of APP [127]. By transfecting SH-SY5Y cells with GST-APP in the presence of YFP-tagged TrkB wild-type or kinase death mutant (K572R), and then treating the cells with BDNF, Xia et al. found that BDNF induced TrkB to phosphorylate APP Y687 residue and APP trafficking to trans-Golgi network, resulting in the decrease of APP exposure to δ-secretase cleavage. Thus, δ-secretase cleaves TrkB, leading to the reduction of p-APP Y687 and alteration of APP trafficking [209]. Moreover, they reported that both TrkB (N365 and N486/489 residues) and APP can be cleaved by δ-secretase in AD brains, resulting in the mitigation of TrkB signaling and the reduction of p-APP Y687. Therefore, both BDNF/TrkB pathway and δ-secretase may be potential targets for AD treatment [210]. The Sortilin Related Receptor 1 (SORL1/SORLA) and its SNP are highly associated with the occurrence and development of late-onset AD and have been shown to affect the metabolism, trafficking, and processing of APP [211–213]. BDNF activates the transcription of *Sorla *via the ERK pathway, thereby diminishing the production of Aβ [214]. On the other hand, *Sorl1*-knockout mice exhibit lower levels of BDNF and fewer deposits of Aβ in the brain [215]. SORL1 inhibits the degradation of APP by γ-secretase, resulting in the reduction of toxic Aβ. Moreover, the expression of BDNF is decreased via the SORL1–NMDAR–CREB–BDNF signaling pathway [216]. These findings suggest that the beneficial effects of BDNF on APP processing are at least partly dependent on SORL1. However, in human pluripotent stem cells, depletion of SORL1 contributes to AD by selectively impairing the neuronal endosomal trafficking of APP, which is independent of APP processing [211]. This discovery seems to echo the sentiment that risk factors for late-onset AD may be characterized moreso by deficits in trafficking and clearance than production and processing.

It is worthwhile to mention that another neurotrophin, nerve growth factor (NGF), has been shown to regulate APP processing via an independent set of receptors (TrkA and p75^NTR^) and sortilin [217]. Advanced Aβ-amyloidosis is characterized by the impaired metabolism of NGF and a concomitant loss of cholinergic synapses and neuronal phenotype in the basal forebrain of McGill-R-Thy1-APP transgenic rats [218]. This suggests that deficits in NGF metabolic signaling may contribute to the high vulnerability of cholinergic neurons in AD. There is also a difference in BDNF and NGF signaling to regulate APP processing. The APP-TrkA binding sites encompass both α- and β-secretase cleavage sites. When NGF binds to TrkA, it may drive APP metabolism in a manner that promotes processing via the non-amyloidogenic pathway [219]. The phosphorylation of APP at Threonine 668 (T668) increases the gene expression of BACE1 [220]. NGF blocks the T668 phosphorylation of APP and promotes the normal metabolism through TrkA signaling [221, 222]. NGF promotes the binding of TrkA to APP, thereby hindering the interaction between APP and BACE1. The NGF/TrkA/APP pathway is linked to the Tyr kinase signaling adaptor SH2-containing sequence C [221]. NGF binding with TrkA can mediate the phospholipase C-γ (PLC-γ) [223], ERK [224], and PI3K/Akt signaling pathways [225]. TrkA and p75^NTR^ receptors share the same binding site in the APP juxta-membrane domain [226]. APP (597–695) is necessary for the interplay between APP and p75^NTR^ [226]. The binding of sortilin to TrkA promotes TrkA anterograde axonal transport, strengthens neurotrophic factor signal transduction, and interacts with APP to affect its metabolism [227].

NGF is essential for the survival of cholinergic neurons, and it is a potential therapeutic target for AD. Results of a phase 1/2 clinical trial demonstrated that while delivering adeno-associated virus (AAV)-*NGF* into the cholinergic neurons of the nucleus basalis of Meynert of AD patients is safe, it has no benefit on cognitive improvement [228]. However, a follow-up analysis on the autopsied brains of three trial participants revealed that NGF failed to reach the cholinergic neurons in any of the injections. Therefore, further studies are needed to determine the clinical efficacy of NGF gene therapy [228]. Tuszynski et al. also reported that the *BDNF* gene therapy might be better than NFG in AD treatment [229]. BDNF is widely expressed in the cortex and is more potent than NGF to rebuild neural circuits, ameliorate cell loss and improve neuronal function in AD. Additionally, targeted delivery of the *BDNF* gene into the entorhinal cortex or hippocampus may be more effective for AD treatment [230].

### Interaction with inflammatory factors

Lipopolysaccharide (LPS) is an endotoxin from the outer membrane of Gram-negative bacteria. Direct injection of LPS into the brain or periphery is a popular method used to study and induce inflammation that activates both the neuroimmune and neuroendocrine systems [231]. Administration of either pro-inflammatory cytokines or LPS leads to a remarkable decrease in *BDNF* gene expression [232]. The neuroinflammation- and LPS-induced memory deficits have been attributed to the activation of TLR4/NF-κB signaling and inhibition of CREB/BDNF expression in AD models [233]. Inflammation significantly decreases BDNF transcription. A single intraperitoneal injection of *E.* *coli* has been shown to profoundly reduce the expression of different *BDNF* transcripts in the hippocampus of aged rodents [234]. More specifically, aged rats exhibit a loss of the exon IV-specific transcript in CA1, exon II- and VI-specific transcripts in CA3, and exon I- and II-specific transcripts in the dentate gyrus [234]. These effects may be mediated by C/EBPβ, an inflammatory cytokine-activated transcription factor, which has been shown to bind to the *BDNF* promoter and repress its transcription [235]. In turn, BDNF deficiency has also been shown to promote C/EBPβ activation by stimulating the JAK2/STAT3 signaling pathway, indicating that these mechanisms may be coupled together [28]. Importantly, triggering this cascade either via BDNF depletion or C/EBPβ activation could accelerate Aβ and tau pathology in 3× Tg mice, suggesting that BDNF/TrkB reduction and C/EBPβ activation may work cooperatively to drive AD pathogenesis. Although BDNF links inflammation and neuroplasticity, the systemic inflammatory response affects not only BDNF but also NGF and neurotrophin-3 (NT-3) [232]. More evidence is needed to determine how inflammation specifically alters the transcription of *BDNF* and the underlying mechanisms.

Our previous studies have confirmed that the expression of NF-κB is increased in the brains of AD patients, and that NF-κB signaling up-regulates human BACE1 gene transcription to facilitate β-secretase cleavage and Aβ generation (Fig. 2) [15]. Furthermore, we have shown that the GSK3β-mediated BACE1 gene expression is dependent on NF-κB signaling, and that inhibition of GSK3β can decrease BACE1 expression and reduce Alzheimer-associated phenotypes [236]. The sAPPβ has also been shown to activate NF-κB, resulting in the production of inflammatory cytokines (i.e., IL-6) in microglial cells and hippocampal neurons [237]. Collectively, these data suggest that the NF-κB-mediated Aβ production and neuroinflammation may be potential targets for AD treatment. To that end, a few key points regarding the interaction between BDNF and NF-κB in AD should be kept in mind. First, since the *BDNF* gene contains binding sites for activated NF-κB in the 5’ flanking region of exon IV, NF-κB plays an important role in BDNF-induced neuroprotection [40, 238]. Specifically, activated NF-κB can translocate into the nucleus, where it binds to the promoters on transcripts I, III and IV of the *Bdnf* gene to initiate BDNF transcription [53, 238–240]. Second, exogenous BDNF promotes the TrkB-mediated NF-κB activation, which is beneficial for neuronal survival [238]. BDNF treatment has been shown to dose-dependently increase the mRNA and protein expression of Bcl-xL in the rat hippocampus through phosphorylation of NF-κB at the Ser529 site and the activation of casein kinase II [241]. Alternatively, blocking NF-κB activation suppresses BDNF-induced late-phase LTP [242]. The crosstalk between BDNF and NF-κB is critical for neuroprotection. However, chronic NF-κB activation will lead to neuroinflammation, followed by neurodegeneration and cognitive impairment. Further examination of the neuroprotective concentrations of BDNF and the period of NF-κB activation is warranted. These findings would provide key insights into the clinal relevance of BDNF-targeting therapies in AD.

## BDNF-targeting strategies for AD modification

**Fig. 3:**
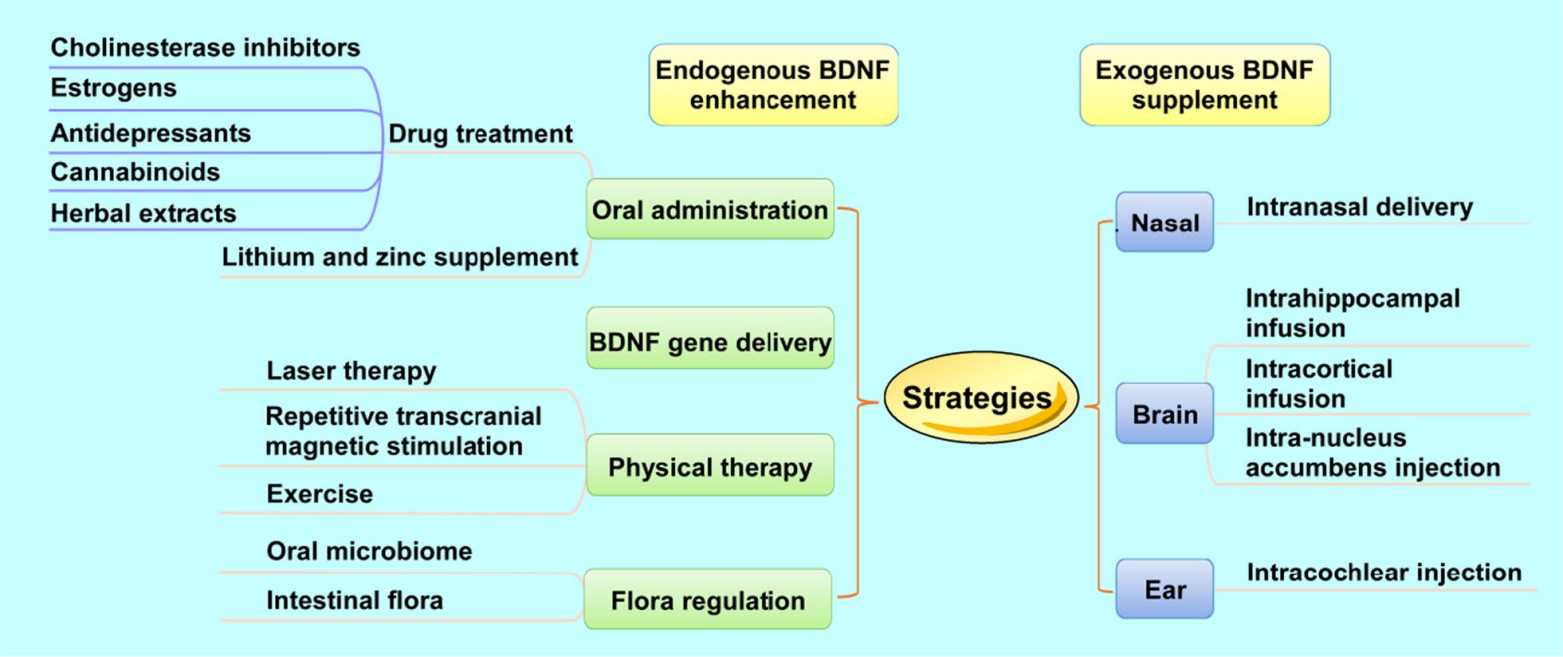
Strategies to improve BDNF levels in the brain. The current therapeutic approaches to enhancing  BDNF concentration include endogenous BDNF enhancement and exogenous BDNF supplement. The former one aims to induce endogenous BDNF production or secretion. The latter one attempts to release BDNF in situ or further transport it into target brain regions

Numerous studies have suggested that therapeutically increasing BDNF levels in brain regions important for memory and cognition may lead to improved clinical outcomes of AD patients [183, 243]. However, the delivery route of exogenous BDNF is limited due to its short plasma half-life and the limited diffusion across the blood–brain barrier (BBB) [244–246]. As a result, many intervention strategies have sought to restore BDNF level and signaling endogenously. These therapies target BDNF either by directly promoting its endogenous production (i.e., via *BDNF* gene therapy) or indirectly enhancing BDNF signaling and secretion in the brain (i.e., via exercise). In the following section, we further discuss the current therapeutic approaches to targeting BDNF in the treatment of AD (Fig. 3). Several review papers have already presented a comprehensive overview and analysis of the outcome of clinical trials involving various BDNF-targeting pharmacological treatments in neurodegenerative diseases [247–249]. Therefore, we specifically focus on providing novel insights into the molecular mechanisms underlying current BDNF-targeting therapeutic strategies in AD. We will explain how recent preclinical and clinical research findings have inspired new approaches to administering or modulating BDNF signaling, and the potential of BDNF as a diagnostic biomarker of or a therapeutic agent for AD.

### Improvement of endogenous BDNF production

#### Drug treatment

Currently, the FDA-approved drugs for AD include acetylcholinesterase inhibitors (AChEIs), an NMDAR antagonist, and the IgG1 anti-Aβ monoclonal antibody (aducanumab). Approved AChEIs—including donepezil, galantamine, and rivastigmine, and the approved NMDAR antagonist (memantine) are symptomatic treatments that do not treat the underlying pathological cause of AD. Thus, aducanumab is the first and only disease-modifying drug licensed for AD [6]. Most (if not all) of the drugs approved for AD treatment are known to influence the level of BDNF. In the following section, we will discuss molecular mechanisms underlying the association between BDNF signaling and drugs that have been approved for the treatment of AD.

**AChEIs**

A pathological hallmark of AD is that the cholinergic neurons of the basal forebrain are the first to fall prey to neurodegeneration [250]. AChEIs such as donepezil enhance cholinergic transmission and have been approved for the treatment of AD on the basis that they were found to delay the progression of cognitive decline in clinical trials. Notably, experimental studies have also shown that AChEI administration enhances the cholinergic tone in cholinergic neurons of the basal forebrain in mice, and that these effects are mediated by the activation of Trk receptors [251]. Similarly, BDNF promotes the survival and differentiation of cholinergic neurons in the same region of the rat brain [252]. These findings suggest that AChEI administration may have some neuroprotective effects in AD, which is conferred by the activation of neurotrophic signaling. In support, clinical studies have shown that the AChEI donepezil increases the level of CNS BDNF in AD patients [208].

As the neuroprotective effects of AChEIs are transient at best, a more provocative question is what mechanism governs their regulation of neurotrophin signaling. One possible explanation comes from studies on the effect of AChEIs in other neurodegenerative conditions. Administration of donepezil has been found to protect against vascular dementia by inhibiting the nuclear translocation of histone deacetylase 6 (HDAC6) and the binding of HDAC6 to *BDNF* promoter IV, which enhances *BDNF* expression [253]. HDAC6 is upregulated in the cortex and hippocampus of AD patients [254, 255]. The consequences of HDAC6-BDNF binding have previously been studied in the context of other risk factors for AD. For example, ApoE4 has been shown to promote the nuclear translocation of HDACs in human neurons, resulting in decreased BDNF expression [255]. Specifically, ApoE4 has been found to induce HDAC6 to bind to *BDNF* promoter IV, thereby inhibiting the expression of BDNF. Therefore, these findings suggest that inhibiting the HDAC6-BDNF binding in the cortex could increase BDNF levels and exert neuroprotective effects in AD. Another key question is what BDNF signaling pathways do AChEIs activate. Previous experimental studies have shown that administration of donepezil  or galantamine in mice enhances the production of BDNF, thereby suppressing neuronal apoptosis via the activation of PI3K/Akt and ERK pathways and phosphorylation of CREB [256]. However, it is worth re-stating that the neuroprotective effects of AChEIs do not prevent the progression of AD. Therefore, although these studies suggest that AChEIs can exert neuroprotective effects via enhancing endogenous BDNF levels, more investigations are required.

***Antidepressants***

Depressive symptoms are common in patients with cognitive impairment. The overall prevalence of depression in AD patients is up to 50% [257–259]. A large-scale longitudinal study has found that the depressive symptoms in AD patients reflect prodromal features of dementia, and dementia is not likely a consequence of long-term depression [260]. This suggests that the pathological mechanisms may differ from those of depressive symptoms in adulthood–that is, in adults without dementia. Despite these differences, antidepressants are still the only treatment option available for the depressive symptoms in dementia[261]. In general, the effect of antidepressants on BDNF expression is not well understood. Several studies suggest that antidepressants like the selective serotonin reuptake inhibitor (SSRI) fluoxetine increase BDNF levels and are dependent on normal TrkB signaling to elicit their behavioral effects [262, 263]. This implies that the therapeutic efficacy of SSRIs may be dependent upon activation of the BDNF/TrkB pathways. However, other studies have reported that certain SSRIs (i.e., fluoxetine, paroxetine, and sertraline) regulate the expression of BDNF mRNA in a dose- and time-dependent manner, such that the acute treatment downregulates BDNF expression, whereas chronic treatment upregulates it [264, 265]. One possible explanation for this effect is that the bi-phasic shifts in BDNF regulation may be caused by differences in the expression pattern of individual BDNF exons. For example, 4 h after systemic injection of paroxetine, the expression of *BDNF* exon IV was found to be selectively downregulated in the rat hippocampus [266]. In rats, neuronal activity has been shown to induce *BDNF* exon IV expression as an immediate-early gene response, meaning *BDNF* exon IV mRNA levels can exhibit fast and transient changes, whereas *BDNF* exon I levels exhibit slower responses [266, 267]. Moreover, the therapeutic effects of paroxetine therapy are associated with polymorphism of the *BDNF* gene, whereby carriers of the A allele of *BDNF* G196A polymorphism respond better to the paroxetine therapy in AD-related depression [268]. These findings support the notion that the ability of SSRIs to alleviate depression-related symptoms may be mechanistically linked to the BDNF/TrkB signaling. Nonetheless, although antidepressant drugs are a primary therapeutic approach currently used for the treatment of depression in AD patients, several systematic meta-analyses have suggested that SSRIs fair no better than a placebo in their ability to alleviate depressive symptoms in AD [269–272]. Additional high-quality randomized controlled trials with different drug types, dosages, and treatment periods should be conducted to confirm the effectiveness and safety of antidepressants in AD patients.

***Estrogens***

Estrogen and its receptor-mediated signaling pathways play vital roles in brain function. Both estrogen and BDNF have been shown to exert highly potent effects in the hippocampus, and thus have been explored as potential pathological mediatory and therapeutic targets in psychiatric conditions characterized by memory loss [273–275]. Estradiol (E_2_) and BDNF have also been shown to help regulate many of the same biological functions, including modulating the activity of NMDARs (especially the NR_2B_ subunit), promoting neurogenesis in the dentate gyrus, and facilitating the formation of memories [276, 277]. It has been reported that estrogen receptor α (ERα) and BDNF are colocalized in CA3 subregion of the developing hippocampus [278, 279]. LPS-induced sickness behavior in mice shows that the role of BDNF in the response to neuroinflammatory challenge occurs in a sex-dependent manner [280]. Notably, LTP was found to produce an elevated inflammatory response in the cortex and hippocampus of wild-type males, as well as in BDNF^+/−^ males. Alternatively, the elevated inflammatory response was found to occur only in BDNF^+/−^ females (not in wild-type females) and only in the hippocampus. These results either suggest that the BDNF/TrkB signaling may be significantly more sensitive to inflammatory insults in the female hippocampus, or that the basal levels of BDNF are significantly higher in the hippocampus of females than males.

Inherent differences in the role of BDNF as an inflammatory mediator between males and females may arise because the *BDNF* gene contains a sequence homologous to the estrogen response element [281], and the estrogen ligand-receptor complexes can bind to this sequence and rapidly increase BDNF transcription. Additionally, the aromatization of testosterone in male mice leads to high levels of E_2_ in the brain [282]. As a result, the expression of BDNF can still be regulated through estrogen-mediated mechanisms in male mice [280]. However, the effects of exogenous E_2_ treatment on various types of memory, and the estrogen-receptor pathways that are activated, have been shown to differ significantly in the hippocampus of male and female rodents [283]. Thus, these differences may be more related to the inherent differences in the expression of estrogen-receptors and downstream signaling pathways between males and females than to E_2_. In agreement, BDNF may act as a signaling molecule downstream of E_2_ to mediate its structural and electrophysiological effects [284]. E_2_ and BDNF have been shown to share several signal transduction pathways and transcription factors, such as AKT, ERK, MAPK, PI3K, Src/Fyn, Ca^2+^/calmodulin-dependent protein kinase II (CaMKII) and CREB [285–288]. 17β-estradiol administration induces the phosphorylation of TrkB and the expression of mature BDNF. However, 17β-estradiol activates hippocampal TrkB signaling independently of enhanced mBDNF [289]. Although many studies have highlighted the benefits of estrogen replacement therapy (ERT) among AD patients [290–293], the impact of ERT on the risk of cognitive decline remains highly contentious [294, 295].

***Cannabinoids***

Since the 1990s, the endocannabinoid system has received increasing interest due to its neuroprotective effect, and there is considerable evidence suggesting that targeting the cannabinoid system might be an effective strategy to protect against AD [296–298]. Cannabinoid type 1 (CB1) receptors primarily localize at nerve terminals and regulate excitatory and inhibitory neurotransmission [299]. In kainic acid (KA)-induced excitotoxicity, inactivation of CB1 receptors can decrease the KA-induced BDNF mRNA levels, indicating that CB1 receptor-mediated neuroprotection may be, at least partially, dependent on BDNF expression [300]. The CB1 receptor is the main molecular target of endocannabinoids and phytocannabinoids, such as Δ^9^-tetrahydrocannabinol, extracted from the *Cannabis sativa* plant [301]. To better understand CB1/BDNF interaction, healthy volunteers were intravenously injected with Δ^9^-tetrahydrocannabinol, which increased serum BDNF levels [302]. One possible explanation is that the CB1 receptor-mediated BDNF expression relies on the activation of the *BDNF* gene promoter IV via the PI3K/Akt/mTORC1/BDNF pathway, which is capable of enabling rapid responses to promote BDNF production [303]. A major drawback of using Δ^9^-tetrahydrocannabinol as a therapeutic agent in AD is that it has been shown to produce deficits in cognitive behaviors that are impaired in AD, such as learning and memory [304]. However, overexpressing BDNF in these regions protects against the cognitive deficits induced by adolescent cannabis exposure in mice [304]. In turn, BDNF-TrkB-CB1R interactions promote the release of endocannabinoids at cortical excitatory synapses [305]. Endogenous BDNF also plays a crucial role in cannabinoid-induced neurogenesis in the subventricular zone and hippocampal dentate gyrus [306]. Although cannabinoids have demonstrated the potential to offer multifaceted protection against AD, further studies are warranted to determine whether chronic administration of cannabinoids can be considered a safe, effective, and low-cost therapy for AD.

***Herbal extracts***

Herbal extracts have been proposed as an alternative medicine to delay the progression of AD, and some extracts have been shown to work through regulating BDNF. For example, resveratrol (3, 5, 4’-trihydroxy-*trans*-stilbene) treatment ameliorates oxidative stress and cognitive deficits in a rat model of vascular dementia by increasing hippocampal BDNF expression [307]. Chronic administration of curcumin, the main active ingredient in turmeric, alleviates AD-associated cognitive impairments via upregulating BDNF/ERK and Akt/GSK3β signaling in the hippocampus [308–311]. However, as the low bioavailability of curcumin limits its effect in humans, some modified curcumin formulations are being studied. Huperzine A is a novel lycopodium alkaloid extracted from the Chinese herb *Huperzia serrata* (Qian Ceng Ta). It belongs to the class of non-competitive AChEIs, and has an antagonistic effect on NMDARs [312]. Huperzine A improves oxidative glutamate toxicity by activating the BDNF/TrkB-dependent PI3K/Akt/mTOR signaling pathway [313]. Moreover, oral administration of huperzine A remarkably alleviates the neuronal damage and memory deficits by increasing the expression and levels of BDNF, which it accomplishes by phosphorylating the MAPK/ERK pathway [314]. However, in a recent phase II clinical trial in individuals with AD, huperzine A (200 μg) failed to demonstrate clinical efficacy [315]. Other herbs, such as *Ginkgo biloba*, *Panax ginseng*, *Rehmannia glutinosa* Libosch., *Polygala tenuifolia* Willd, *Salvia miltiorrhizae* Bunge, and *Ficus erecta* Thunb. leaves, have also been investigated for therapeutic efficacy in AD and are considered as potential agents that could endogenously increase BDNF [316–323]. However, clinical evidence supporting the beneficial effect of herbal extracts on BDNF is still lacking.

#### Lithium and zinc

Lithium or zinc supplementation has been proposed as a novel AD therapeutic strategy due to their modulatory effects on multiple targets, including inflammation, autophagy, oxidative stress and mitochondrial dysfunction [324–327]. Notably, lithium treatment in AD patients has been shown to increase BDNF serum values (~ 30%) and mitigate cognitive impairment [328]. However, a negative correlation between lithium in drinking water and changes of AD mortality has been reported [329]. It should be noted that limitations in the experimental design may have caused these conflicting results. While using “microdoses” of lithium in mild cognitive impairment has yielded encouraging results, prolonged exposure and high doses of lithium treatment induce toxicity [330, 331]. For example, De-Paula et al. stimulated primary cortical and hippocampal neurons with therapeutic (2 mM) and subtherapeutic (0.02 and 0.2 mM) dosages of lithium [332]. They found that administering low subtherapeutic doses of lithium (0.02 mM) had a more extensive and robust effect on enhancing neuronal BDNF in different brain regions than the higher doses typically considered to be therapeutic. Interestingly, the role of lithium on BBB integrity in rats is dependent on their state of mental health. Whereas lithium treatment repairs the stress-induced BBB hyperpermeability in the hippocampus, it has the opposite effect in normal controls [333]. This suggests that lithium may interact with BDNF signaling pathways in a context-dependent manner.

Experimental research has shown that zinc interacts with multiple AD-related pathologies, some of which are directly mediated by BDNF. Zinc activates GPR39 metabotropic receptors in the CNS [334, 335]. GPR39 knockout mice display decreased CREB and BDNF levels in the hippocampus, but not in the frontal cortex [336]. This suggests that the expression of BDNF and CREB can only be modulated by zinc in certain brain regions. In zinc transporter-3 knockout mice, deficits in learning and memory were observed at 6 months of age, accompanied by decreased levels of TrkB, NMDAR2b, α-amino-3-hydroxy-5-methyl-4-isoxazolepropionic acid receptor (AMPAR)2a, BDNF, and pro-BDNF [337]. Oral supplementation with zinc has been found to reduce Aβ and tau pathology in the hippocampus, ameliorate mitochondrial dysfunction, reduce inflammation, inhibit oxidative stress, and increase BDNF concentration [338–343]. Importantly, zinc gluconate solution can cross the BBB to biosynthesize fluorescent zinc oxide nanoclusters, enabling high spatiotemporal bioimaging [344]. Therefore, zinc supplementation has the potential to play a dual role in AD treatment, neuroprotection and bioimaging, with the latter function being beneficial for evaluating its own efficacy. Results from nuclear magnetic resonance spectroscopy, light scattering, and cryo-electron microscopy indicate that Zn^2+^ binding to the BDNF Met66 prodomain and Val66 prodomain result in different conformational and macroscopic structures [345]. The substitution of Met66 results in a higher affinity of prodomain to Zn^2+^, owing to the His40-mediated stabilization of its multimeric structure. Moreover, the molecular mechanism of zinc deficiency-induced cognitive impairment is associated with hippocampal BDNF DNA methylation [346]. In brief, this suggests that the upregulation of BDNF may contribute to the neuroprotective effects of lithium or zinc in AD treatment.

#### BDNF gene delivery

The primary obstacle for *BDNF* gene delivery is the selection and optimization of vehicles. Gene-delivery vehicles are mainly divided into two categories: synthetic carriers and recombinant viruses. The former includes polymers and liposomes, and the latter includes AAV, poxvirus, retrovirus, adenovirus, lentivirus and herpes simplex virus [347, 348]. Each delivery vector has its advantages and disadvantages. Polymer-based vectors used for *BDNF* gene delivery include nanoparticles and hydrogels, among others [349]. Liposomes, which are natural biodegradable lipid bilayers, have great advantage of being similar to natural cell membranes. These nonviral carriers are based on the electrostatic interactions of cationic compounds that spontaneously complex with the BDNF plasmid. Polymer-based vectors exhibit a number of desirable traits, including ease of manufacturing, good safety and stability, low immunogenicity, and simple methods to incorporate target ligands [350, 351]. Unfortunately, the transfection efficiency of polymers as gene-delivery vectors is several orders of magnitude lower than that of recombinant viruses. Thus, using a recombinant virus is still the primary means for *BDNF* gene delivery [352, 353]. On the downside, viral vectors can induce inflammation and immune responses. Although the systemic immune response induced by systemic injection of viral vectors can be considered harmful in clinical trials, gene therapy of the brain is considered a relatively safe intervention strategy [354, 355].

*BDNF* gene delivery exerts protective effects against Aβ- and tau-related pathologies in AD. However, this treatment has no direct action on Aβ deposition and tau hyperphosphorylation. Treating J20 APP transgenic mice with Lenti-BDNF gene delivery for 5 months alleviated learning and memory deficits, ameliorated synaptic degeneration, and reduced atrophy [126]. However, this BDNF treatment did not change amyloid plaque density. Similarly, P301L mutant tau transgenic mice receiving recombinant human *BDNF* gene using an AAV8 vector (AAV-BDNF) showed higher BDNF levels in the brain and improved memory deficits, although the AAV-*BDNF* gene delivery had no direct effect on tau protein, GSK3β, and phosphatase PP2A [189]. On the other hand, BDNF supplementation indeed did successfully alleviate tauopathy-induced memory impairments by inhibiting neuron loss, synaptic degeneration, and impaired neurogenesis [189].

FDA-approved clinical trials of gene therapies have previously applied the AAV delivery strategy because it can target specific neurons in the brain regions, allowing widespread and stable expression of proteins with the safety of long-term treatment [356–358]. MR-guided infusion of AAV2-BDNF has been used to accurately and consistently target BDNF into the non-human primate entorhinal cortex [230]. Moreover, real-time MR imaging of AAV in the primate brain has been applied to accurately target intracranial structures and monitor the vector distribution in real-time during injection, thereby ensuring accurate targeting and spread of the vector [359]. Mutant AAVs have also been studied intensively. Delivery of BDNF using the tyrosine triple mutant AAV (tm-scAAV2) showed that the RNA expression of BDNF was about 300 times higher than that of the AAV group, and produced significantly higher proteins [360]. These methods enable more effective clinical translation to alleviate neuronal loss and prevent neuronal dysfunction in AD. In February 2021, a first-in-human Phase I clinical trial was launched to assess the safety and efficacy of modified AAV2-BDNF in the treatment of patients with AD or MCI [361]. The modified method for delivering BDNF will be more conducive for the delivery and distribution of BDNF into the entorhinal cortex and hippocampus.

Another approach for extended delivery of BDNF is the use of cell-based vectors, such as neural stem cells (NSCs), mesenchymal stem cells (MSCs), Schwann cells, CD4 T cells, and fibroblasts [362–365]. Direct *BDNF* gene delivery using MSC can overcome BBB blocking [366]. In previous research, BDNF-transduced bone marrow stromal cells (BMSCs) were transplanted by intravenous injection into irradiated female SJL/J mice for 8 weeks, resulting in a dramatic delay of experimental autoimmune encephalomyelitis onset and a reduction in overall severity [367]. On the other hand, these BDNF-producing cells only allow prolonged delivery of BDNF. Unfortunately, this method is difficult to be controlled precisely because the delivered BDNF dosages are dependent on cell survival and the stability of transfection. Another concern is that bone marrow-derived cells can migrate and reside in various nonhematopoietic tissues, therefore producing undesired effects. Thus, encapsulation of these BDNF-producing cells has been proposed to achieve continuous and local release. Encapsulated BDNF-producing fibroblasts in alginate-poly-*L*-ornithine survived for at least one month after being transplanted into the site of cervical spinal cord injury in rats without immunosuppression [363]. Transfection of *BDNF* gene recombinant MSCs via the adhesive peptide PPFLMLLKGSTR-modified scaffold improved cell survival and BDNF expression [368]. Alginate-based compositions have also been used to transport NSCs-BDNF and BMSCs-BDNF, maintaining long-term survival and proliferation of cells, as well as controlled release of BDNF [362]. However, when delivering the *BDNF* gene to APP transgenic mice after “disease onset”, no protection against neuronal death was found following a 1.5-month therapeutic period [88]. This suggests that *BDNF* gene delivery might not be a suitable therapeutic strategy for AD at all stages of the disease. As such, both early and long-term treatments may be required.

#### Physical interventions

Numerous physical interventions have been used to slow down the progression of AD, such as laser therapy, repetitive transcranial magnetic stimulation (rTMS) and exercise [369–372]. Low-level laser treatment has been shown to alleviate Aβ-induced neuronal loss and dendritic atrophy by enhancing BDNF via ERK/CREB pathway activation [32]. In clinical trials, laser therapy has been successfully applied to treat prostate cancer, lung cancer, and acute pain [373–375]. However, it has not been translated well to AD patients. Novel approaches and more clinical studies are needed to evaluate the efficacy of laser therapy for Alzheimer’s patients. rTMS is a non-invasive therapy for cognitive dysfunction in AD that acts by regulating neuronal excitability [376]. Different frequencies of rTMS target different brain regions, making it theoretically possible to improve cognitive deficits that are highly localized to a particular brain region [377]. Additionally, the cognitive benefits of rTMS have been associated with the induction of hippocampal BDNF expression. Low-frequency (1 Hz) rTMS increased hippocampal BDNF and NMDAR expression, and rescued deficits in LTP and spatial memory in an Aβ_1-42_-induced toxicity rat model [378]. While this approach seems promising, changes in BDNF expression following rTMS treatment are difficult to detect in human brain tissues. The role of transcranial direct current stimulation (tDCS) in memory improvement has also been investigated as a possible intervention strategy that could promote the BDNF signaling pathway [379, 380]. Mice subjected to tDCS stimulation exhibit enhanced acetylation at *Bdnf* promoter I that persists for one week, suggesting that remodeling of *Bdnf* may mediate the long-lasting effects of tDCS treatment. The action of tDCS varies in Val/Val and Met/Met carriers [381]. Compared with BDNF^Val/Val^ mice, BDNF^Met/Met^ show decreased levels of *BDNF* exon IV- and VI-specific transcripts, higher trimethyl-histone-H3-Lys27 binding to *BDNF* exon V, VI and VIII promoters, and impaired trafficking of *BDNF* VI transcript to CA1 and CA3 regions. Moreover, tDCS promotes synaptic plasticity via activity-dependent BDNF secretion [382].

Physical exercise, especially aerobic exercise, is beneficial for improving cognitive function. Studies have attributed many of the therapeutic benefits of exercise in AD to its effect on BDNF levels [383, 384]. Exercise increased the levels of pCREB, CaMKIV and BDNF in the CA1 and dentate gyrus of rats with intracerebroventricular infusion of 250 pmol/day Aβ_1-42_ peptides for two weeks [385]. Four weeks of cardiovascular exercise in mice led to a remarkable increase in BDNF mRNA and protein levels, accompanied by an improved synaptic load in the dentate gyrus region [386]. Moreover, six months of voluntary physical exercise in 5× FAD mice rescued cognitive deficits by increasing astrocytic BDNF in the hippocampus [387]. Astrocyte-released BDNF plays a vital role in modifying the morphology and density of dendritic spines through a truncated form of the TrkB (TrkB T1) receptor [388]. The TrkB T1 receptor specifically localizes at GFAP^+^ astrocytes to increase the number of GFAP^+^ astrocytes and improve Aβ plaque-associated astrocytic morphology via the BDNF/TrkB signaling pathway [386]. A ten-week treadmill training program in APP/PS1 mice also restored hippocampal memory and dendritic arbor in the CA1 and CA3 regions via BDNF/TrkB signaling pathways [389]. For obvious reasons, these results cannot be directly translated to humans. Exercise protocols used in animal studies are significantly different from those used in humans, and how exercise enhances BDNF levels during AD is still unknown. A meta-analysis by da Costa Daniele et al. found that exercise indeed promotes neurogenesis and reduces cerebral Aβ deposition in both healthy and dementia models [390]. However, evidence on exercise-induced inflammation, oxidative stress, metabolism and insulin sensitivity was scarce. Few studies have compared the beneficial effects among acute exercise, chronic exercise and high-intensity training in AD. It has been demonstrated that aerobic exercise training is associated with increased polyunsaturated free fatty acids, decreased phospholipids, sphingolipids and ceramides, and alterations of gut microbiome metabolites–among which, approximate 30% of these metabolites are correlated with altered BDNF levels [391]. Thus, more direct evidence should be obtained to confirm how to use exercise to prevent or treat AD.

#### Regulation of microbiota

A growing body of evidence has suggested that dysregulation of the human microbiome may contribute to the pathogenesis of AD. Poor dental status (i.e., loss of teeth) has been considered an early sign of AD, and irregular tooth brushing is a high risk factor for dementia [392, 393]. *P. gingivalis*, *T. forsythia,* and *T. denticola* have been implicated as the main pathogens responsible for triggering inflammatory responses, and are associated with the pathogenesis of AD [394]. Gut microbial diversity is altered in AD patients [395]. Compared with healthy controls, AD individuals’ microbiome show a lower abundance of *Firmicutes* and *Actinobacteria*, and a higher abundance of *Bacteroidetes* at the phylum level. Researchers have also identified 13 genera as potential CSF biomarkers of AD pathology. Among these, increased levels of *Dialister* and *SMB53* are associated with less AD pathology. The abundance of *Bacteroides*, *Turicibacter* and *SMB53* (family *Clostridiaceae*) is closely linked with CSF chitinase-3-like protein 1 in AD patients, supporting that the change of intestinal bacterial abundance may be correlated with glial activation in AD.

The BDNF level is closely related to the composition of gut microbiota. Compared to mice with normal gut microbiota, germ-free mice show lower mRNA and protein concentration of BDNF in the hippocampus, amygdala and cortex [396–398]. After transferring fecal microbiota, the levels of cognitive behavior, inflammatory mediators, microglia activity, and BDNF in recipient mice are similar to those of donor mice [399]. This mechanism is associated with the activation of AKT-GSK3β/β-catenin pathways. These results suggest that the CNS BDNF levels can be significantly disturbed due to the absence of gut microbiota and restored by microbiota transplantation. Furthermore, probiotic supplements are beneficial for up-regulating BDNF levels. VSL#3 is a probiotic mixture composed of 8 Gram-positive bacterial strains. In aged (20–22 months) male rats, VSL#3 treatment increases the abundance of *Actinobacteria* and *Bacteroidetes*, suppresses microglial activation, and enhances BDNF levels [400]. How might gut microbiota regulate BDNF levels? Some neurochemicals such as neurotransmitters, butyrate, short-chain fatty acids, and secondary bile acids, can be synthesized and recognized by gut microbiota [396, 401–406]. Accordingly, gut microbiota may influence CNS BDNF function by modulating the activity of these neurochemicals.

### Exogenous administration of BDNF

Intravenous injection of BDNF is limited by its short plasma half-life (as short as 0.92 min) and poor BBB permeability [244]. Thus, it is a challenge to evaluate the local distribution and action of BDNF in targeted brain regions. As shown in Table 1, some precise local delivery methods have been proposed, including intra-hippocampal [407], intra-cortical [408–411], intra-nucleus accumbens [412], intranasal [413, 414], and intra-cochlear [415] infusions. Preclinical studies have shown that the brain-specific delivery of BDNF is beneficial for promoting the expression of BDNF receptors, inducing lasting potentiation of synaptic transmission, and increasing neurogenesis and ectopic granule cells [416, 417]. However, exogenous BDNF delivery is hard to apply in clinical settings because most direct delivery methods of BDNF are highly invasive, and treatment duration and dosing times are ambiguous. Moreover, BDNF is unstable and easy to degrade in a biological medium. Intranasal delivery of 70 μg [^125^I]-radiolabeled BDNF results in delivery of 1.6–25.1 ng/ml of BDNF within 25 min in brain parenchyma, and this value increases further by 60 min [418]. In addition to reaching the CNS directly, this concentration of BDNF is sufficient to activate the PI3K/Akt pathway. Thus, a great deal of evidence supports the clinical potential of using intranasal delivery of BDNF because (1) there is a large surface area for drug absorption through the nasal mucosa, (2) intranasal delivery bypasses the BBB, (3) the needle-free and easy self-administration improves patients’ compliance, (4) it enables both rapid and direct CNS delivery of BDNF with high bioavailability by avoiding first-pass hepatic clearance, (5) it causes minimal systemic exposure, (6) a small dosage can be used, avoiding adverse effects, and (7) no drug modification is required. The dosage of intranasal protein is minimal, whereas the administration period is prolonged. Intranasal delivery of BDNF (42 pmol, 1 μM)-PBS solution (bilateral, administered once every two days for a total of seven doses over 14 days) significantly improves the memory performance [419]. In contrast, a higher BDNF dosage (10 μM) does not lead to further improvements, indicating this method has a ceiling effect.

**Table 1:** Local delivery routes of exogenous BDNF

Delivery route	Model	Targeted brain region	BDNF delivery vehicle	Results	References
Intrahippocampal infusion	Rats	Hippocampus	BDNF-containing PBS	Improves lasting potentiation of synaptic function in the dentate gyrus	[416]
		DG	BDNF (no detailed information)	Increases neurogenesis of DG; most new neurons appear to become granule cells	[417]
		vHPC	BDNF dissolved in PBS	Increases excitability in infralimbic targets and supports extinction memories	[411]
		CA1	BDNF-containing sterile saline	Reverses the impairments in memory persistence; generates persistent LTM storage via activation of ERK	[407]
Intracortical infusion	Rats	dmPFC	BDNF-containing PBS	Alleviates cocaine-induced decrease in basal extracellular glutamate; reduces cocaine-mediated increase in extracellular glutamate with the NAc	[408]
				Inhibits cocaine-induced phosphorylation of ERK and CREB	[409]
	Mice	vmPFC	BDNF reconstituted in 0.9% saline	Rescues paradoxical reversal learning enhancement induced by stress or prefrontal cortical damage	[410]
Intra-nucleus accumbens injection	Rats	Nucleus accumbens	BDNF dissolved in saline	Suppresses dopamine release and dopamine-related behaviors induced by methamphetamine	[412]
Intranasal delivery	Rats	Nasal cavity	^125^I-BDNF dissolved in sterile PBS	Intranasal delivery of 70 μg [^125^I]-radiolabeled BDNF results in 1.6–25.1 ng/ml within 25 min in brain parenchyma	[418]
			BDNF reconstituted in sterile PBS	Alleviates cerebral local inflammation induced by ischemia/reperfusion	[414]
			BDNF-containing saline	Improves visual depth perception in amblyopic rats	[413]
Intracochlear infusion	Cats	Cochlear	BDNF-containing sterile artificial perilymph	Increases the total volume of cochlear nucleus to exert neurotrophic effects	[407]
	Guinea pigs		BDNF-containing saline with BSA (1%)	Enhanced survival of spiral ganglion cells	[450]

*BBB* blood–brain barrier, *BDNF* brain-derived neurotrophic factor, *CREB* cAMP-response element binding protein, *ERK* extracellular regulated protein kinases, *SA* self-administration, *TrkB* tyrosine kinase receptor type B, *DG* dentate gyrus, *vHPC* ventral hippocampus, *LTM* g long-term memory, *dmPFC* dorsomedial prefrontal cortex, *NAc* nucleus accumbens, *vmPFC* ventromedial prefrontal cortex, *BSA* bovine serum albumin

Although several reviews and meta-analyses have revealed that the intranasal delivery route is safe and effective [420, 421], there are still some limitations to a carrier-free delivery of BDNF. First, intranasal BDNF delivery can also enter nasal-associated lymphatics and deep cervical lymph nodes [422]. Thus, the effects of intranasal BDNF on the nasal mucosa and the undesired immune response should be examined. Second, simply delivering BDNF in solution is challenging to retain in the nasal cavity due to the fast diffusion from the administered sites and rapid clearance by the mucociliary clearance system [423]. Third, compared with the amount of BDNF applied in the nasal cavity, the amount of BDNF reaching the CNS is small (generally below 1%) [424]. Fourth, some nasal cytochrome P450/proteases may degrade BDNF. Finally, the pharmacokinetic profile of intranasal BDNF must be characterized. Thus, many other carrier-based approaches have been studied for effective nose-to-brain administration of BDNF.

Nanoencapsulation technologies have been widely utilized to solve the limitations of carrier-free delivery of macromolecular drugs. Table 2 summarizes some polymeric nanoparticles used for BDNF delivery. The polymeric nanoparticles are solid colloidal particles in which BDNF can be dissolved, entrapped, encapsulated, or chemically bound to the polymer matrix [425, 426]. PEGylation of BDNF can enhance the diffusion of BDNF in the brain tissue and spinal cord [427, 428]. PEG-based BDNF nano-system, mediated by electrostatic coupling and hydrogen bonding, is beneficial for stabilizing BDNF, protecting against the nonspecific binding with serum proteins, and activating TrkB as well as other downstream signaling pathways [429–431]. Compared with native BDNF, intranasal administration of the nano-BDNF complex can enhance BDNF levels in the hippocampus and brainstem regions by regulating the viscosity and permeability of nasal mucosa [429]. PLGA nanoparticles help to protect drugs from enzymatic degradation and prolong the half-life [432, 433]. To enable sustained local release of BDNF, PLGA microparticles are further patterned with hydrogels [434, 435]. The short-range electrostatic interactions between PLGA and BDNF protein make BDNF adsorb to the surface of nanoparticles rather than encapsulate within the nanoparticles. Meanwhile, the amphiphilic hydrogel polymers enhance the interaction between BDNF and PLGA nanoparticles, resulting in a sustained release for at least 28 days. Therefore, the release profile of BDNF can be regulated by modifying the components of nano-formulations [436].

**Table 2:** Synthetic polymers for BDNF modification or BDNF delivery system

Polymer	Formulation or modification	Preparation	Results	References
PEG	BDNF-PEG^2000^, BDNF-PEG^5000^	Coupled PEG to BDNF carboxyls using carbodiimide	PEG conjugation at the C-terminus of BDNF retains the biologic activity and reduces systemic clearance in vivo	[451]
	BDNF-PEG mixtures	Covalently attached BDNF to PEG	Mixtures with one and two conjugate products maintain high bioactivity in vitro; improve half-life of BDNF in CSF; enhance the penetration into spinal cord tissue	[452]
	BDNF-PEG^2000^-biotin/SA-OX26 (BDNF chimeric peptide)	(1) Attached a hydrazide to one end of PGE and a biotin moiety to the other end; (2) Prepared OX26/SA by thiol-ether linkage; (3) Coupled PEG to BDNF via hydrazide linkers; (4) Conjugated BDNF-PEG-biotin to OX26/SA	The bioactivity of the BDNF chimeric peptide is identical to native BDNF; transported through BBB after intravenous administration; minimizes rapid clearance of BDNF; increases brain uptake of BDNF to about twofold	[453, 454]
	BDNF-PEG^2000^	BDNF in pH 8.0 borate buffer was reacted with PEG	Enhances the diffusion of BDNF into brain tissues	[427]
	BDNF-PEG	N-terminal pegylated form of BDNF	Improves the penetration of BDNF into the spinal cord	[428]
PLGA	BDNF-immobilized PLGA membrane	Incorporated BDNF onto the surfaces of PLGA membrane by heparin immobilization	Controlled release of BDNF for 4 weeks; protects against cavernous nerve; improves angiogenesis in the corpus cavernosum	[455]
PLA	BDNF/PLA macroporous tubular scaffolds (foams)	(1) Dissolved BDNF in BSA solution and then lyophilized to powder; (2) Dispersed BDNF/BSA powder into PLA/DMC solution	PLA tubular macroporous scaffolds with BDNF enhance cell survival and angiogenesis	[456]
Composite materials	PLGA-PLL-PEG microspheres	(1) Conjugated PLGA-PLL-PEG polymer; (2) Dissolved BDNF/BSA into polymer/DCM solution; (3) Fabricated microspheres by double emulsion technique	Yields greater loading and longer-term delivery of BDNF for more than 60 days; maintains the bioactivity of BDNF	[436]
	PLGA microparticles/ PEG hydrogel	(1) Prepared BDNF-loaded PLGA microparticles via water/oil/water emulsion technique; (2) Polymerized PLGA microparticles and PEG hydrogel by UV exposure	Sustained release of BDNF over a period of 56 days; alleviates the reactive glial response; increases the recruitment of astrocytes	[434]

*BDNF* brain-derived neurotrophic factor, *CSF* cerebrospinal fluid, *BBB* brain–blood barrier, *HA* hyaluronic acid, *PEG* polyethylene glycol, *PLL* poly-l-lysine, *PLGA* poly (lac-co-glycolic acid), *SA* streptavidin, *ADSCs* adipose-derived stem cells, *PLA* poly(d,l-lactic acid), *DMC* dimethylcarbonate, *BSA* bovine serum albumin

As derivatives of extracellular matrix (ECM) components, natural biopolymers are advocated to deliver macromolecular drugs and can be adjusted for intranasal drug administration [437]. Collagens are the most abundant proteins to maintain the structural integrity of ECM. BDNF fused with a collagen-binding domain (CBD-BDNF) can specifically bind to collagen [438–440]. Chitosan has similar structural characteristics as glycosaminoglycan, which is the main component of the ECM [441]. As shown in Table 3, collagen and chitosan scaffolds used for BDNF delivery are generally produced on a macroscopic scale. However, native ECM is located in the nanofibrous network structure. To develop biomimetic scaffolds, a collagen-chitosan complex has been made to prepare nanoscale scaffolds [442]. However, no nanoparticles based on collagen or chitosan have been reported for BDNF delivery. Alginate, naturally occurring linear unbranched polysaccharides extracted from brown algae cell walls, has been considered as an ideal biodegradable polymer for continuous delivery of proteins [443]. This is because alginate can be crosslinked by adding divalent cation to the aqueous solution. During the gelation process, proteins can then be incorporated into alginate matrices [444]. As a bioadhesive polymer, alginate can specifically facilitate the delivery to mucosal tissues [445]. Another natural polysaccharide, agarose, is derived from red algae [446]. Upon cooling hot agarose solution in water, a physical crosslinked three-dimensional gel network can be obtained via H-bonding and hydrophobic interactions [447]. Interestingly, proteins such as BDNF exhibit various degrees of H-bonding and hydrophobic interactions [448]. Therefore, agarose has been used as a good coupling partner for loading and delivering BDNF without inflammatory or immunological responses. As shown in Table 3, the alginate- and agarose-based hydrogel system used for BDNF delivery is characterized by sustained release of BDNF, protects neuronal functions and minimizes inflammatory damage. Thus, alginate and agarose hydrogel scaffolds have been used for BDNF-producing cell transplants [362, 366, 449]. In vivo, these encapsulated BDNF-producing cells can release bioactive BDNF, which persists in the injured site over one month and promotes host axon growth. Accordingly, the intranasal delivery and biodegradable nanocarriers may help the development of AD therapy by targeting BDNF. To improve the availability of exogenous BDNF therapy, important questions should be answered concerning the noninvasive transport routes, the therapeutic doses of BDNF, and the safety and clinical efficacy of administering BDNF to AD patients.

**Table 3:** Natural polymers and drug delivery systems for BDNF

Polymer	Formulation or modification	Preparation	Results	References
Collagen	Collagen scaffold-BDNF complex	(1) Prepared LOCS from bovine aponeurosis; (2) Fused CBD to BDNF; (3) Linked CBD-BDNF to LOCS	CBD-BDNF can bind to collagen and concentrate at the injury site; promotes neuronal regeneration and locomotion recovery	[457]
			Reduces cell loss and decreases apoptosis	[438]
			Promotes axonal regeneration; Increases functional nerve growth; Enhances neuronal re-myelination	[458]
Chitosan	Chitosan scaffolds	Cross-linked BDNF to chitosan scaffolds by genipin	Maintains a 30-day-period release; Induces tissue regeneration after traumatic brain injury	[459]
Alginate	Calcium alginate hydrogel	(1) Mixed BDNF into sodium alginate; (2) Dropped CaCl_2_ to form hydrogel microbeads or microspheres	Sustained release of BDNF over 48 days; Promotes axonal regeneration in vivo; Alleviates neuropathic pain	[460]
			Controlled release of BDNF for more than 7 days; Improves depressive-like behavior	[461]
	Cell-seeded alginate hydrogel scaffold	(1) Placed sodium alginate solution in a cylindrical aluminum mold; (2) Cross-linked with Sr^2+^ or Zn^2+^; (3) Socked BMSCs-BDNF suspensions into the hydrogel scaffolds	Releases BDNF from the scaffolds; BMSCs survive in the alginate hydrogel channels; Guides axons to orient parallel to the hydrogel channel and promoted axons growth	[462]
Agarose	Agarose scaffolds	(1) Loaded BDNF into lipid microtubules and mixed with agarose solution; (2) Prepared agarose in situ gel via cooled nitrogen gas	Encourages neurite growth into the scaffolds; Reduces inflammatory response induced by agarose; Enhances regeneration after spinal cord injury in vivo	[463, 464]
		Fabricated scaffolds by freeze-dry processing	Maintains high stability and biocompatibility for at least 1 month in vivo; Supports the growth of injured axons	[465]
	Cell-seeded agarose scaffolds	(1) Fabricated agarose scaffolds and PS fibers to form multi-channels; (2) Filled BMSCs into scaffolds	Secretes BDNF from the scaffolds; Supports host axon regeneration across the lesion gap	[366]

*BDNF* brain-derived neurotrophic factor, *CBD* collagen-binding domain, *HP-β-CD* hydroxypropyl-β-cyclodextrin, *LOCS* linear-ordered collagen scaffold, *BMSCs* bone marrow stromal cells, *PS* polystyrene

## Conclusion

BDNF is a key neurotrophic molecule that has been shown to enhance synaptic plasticity and improve learning and memory. Disruption of BDNF has been found in different stages of AD. In this review, we discuss the effect of BDNF on AD-related pathologies, including Aβ accumulation, tau phosphorylation, neuroinflammation, neuronal apoptosis, and cognitive decline. BDNF/TrkB and the downstream cell signaling pathways, including PI3K/Akt, ERK/CREB, and PKC/GSK3, are further discussed for their effects on AD. Although some data reported that BDNF did not affect AD, higher BDNF levels indeed reduced the risk of AD. Most AD drugs currently used in clinical (e.g. donepezil, galantamine, rivastigmine and tacrine) and many therapeutic agents under development increase BDNF biosynthesis. Therefore, even though BDNF is not the primary molecular target of these drugs, we should not lose sight that BDNF is implicated in the mechanism of cognitive improvement. Many strategies have also been reported to support the possibility that exogenous BDNF supplementation would be an alternative option to improve cognitive function in AD. Biodegradable nanocarriers combined with intranasal delivery of BDNF to avoid invasive administration and improve brain-targeted distribution may provide novel promising approaches for AD therapy.

BDNF plays several vital roles in most neural cells and peripheral systems. In addition to AD, it is also involved in several metabolic syndromes, including atherosclerosis, hypertension, hyperglycemia, type 2 diabetes mellitus, and many other neuropsychiatric diseases such as depression, Parkinson’s disease and Huntington’s disease. More in-depth studies are needed to understand the role of different isoforms of BDNF, and the relationship between peripheral and brain BDNF under pathological conditions.

## Data Availability

Available upon request.

## Abbreviations

AD: Alzheimer’s disease
Aβ: Amyloid β
APP: Amyloid β precursor protein
BDNF: Brain-derived neurotrophic factor
NMDAR: N-methyl-D aspartate receptor
p75^NTR^: P75 neurotrophin receptor
TrkB: Tropomyosin receptor kinase B
JNK: C-Jun N-terminal kinase
JAK2: Janus kinase 2
STAT3: Signal transducer and activator 3
IRAK: Interleukin-1 receptor-associated kinase
NF-κB: Nuclear factor-κB
TLR4: Toll-like receptor 4
MyD88: Myeloid differentiation primary response gene 88
TNF-α: Tumor necrosis factor-α
IL-6: Interleukin-6
MEK: Mitogen-activated protein kinase kinase
ERK1/2: Extracellular signal-regulated protein kinase 1/2
CREB: CAMP-response element binding protein
(C/EBP)β: CCAAT/enhancer binding protein β
PI3K: Phosphoinositide 3-kinase
Akt: Protein kinase B
PLCγ: Phospholipase C_γ_
GSK3β: Glycogen synthase kinase-3β
Cyt C: Cytocheome C
